# Nursing technicians’ perceptions about the use of play and playfulness in professional practices

**DOI:** 10.1590/1980-220X-REEUSP-2025-0024en

**Published:** 2025-07-14

**Authors:** Gabriele Petruccelli, Monika Wernet, Aline Oliveira Silveira, Edmara Bazoni Soares Maia, Cristiane Leite de Almeida, Fernanda Manuela Loureiro

**Affiliations:** 1 Universidade Federal de São Carlos, Programa de Pós-Graduação em Enfermagem, São Carlos, SP, Brazil.; 2 Universidade Federal de São Carlos, Departamento de Enfermagem, São Carlos, SP, Brazil.; 3 Universidade de Brasília, Departamento de Enfermagem, Brasília, DF, Brazil.; 4 Universidade Federal de São Paulo, Escola Paulista de Enfermagem, São Paulo, SP, Brazil.; 5 Escola Técnica Estadual Paulino Botelho, São Carlos, SP, Brazil.; 6 Universidade Católica Portuguesa, Lisboa, Portugal.

**Keywords:** Pediatric Nursing, Hospitalization, Child, Play and Playthings, Licensed Practical Nurses

## Abstract

**Objective:**

To understand nursing technicians’ perceptions in a pediatric inpatient unit regarding the use of play and playfulness in their professional practices.

**Method:**

An exploratory study with a qualitative approach, supported by the Symbolic Interactionism theoretical framework, developed in a pediatric unit of a university hospital in the countryside of São Paulo. Data collection took place from March to December 2024, through semi-structured interviews, with 14 participants. Reflective thematic analysis supported data assessment.

**Results:**

The topics “Purposes of adopting play and playfulness” and “Determinants of adopting play and playfulness” revealed that nursing technicians link resources with the execution of technical procedures and formation of bonds. However, they highlighted a context that was not very supportive of this use and that led to the meaning that these resources were other professionals’ responsibility.

**Conclusion:**

The use of play and playfulness predominated for the execution of procedures, with a secondary, informal, voluntary and non-institutionalized place in nursing care for children.

## INTRODUCTION

Hospitalization is a complex event for children that generates stress, anxiety, suffering, anguish and tension, with the potential to become a traumatic experience. Moreover, both the child and the person accompanying them are exposed to the unknown as their daily lives are changed^(1,2)^.

Furthermore, during the period of hospitalization, patients may be exposed to potentially painful diagnostic and therapeutic procedures as well as being separated from the family environment, friends and educational space^(1,3)^.

In this scenario, it is common to face restrictions on playing, an essential occupation^(1,3)^. The incorporation of play and playfulness is noted as transforming hospital environments, especially because it symbolizes family to children^(3,4)^, bringing a sense of normality and continuity to their life. This makes these resources act as an escape valve, helping in the development of strategies to cope with illness and hospitalization, favoring distractions and entertainment, facilitating information assimilation, and reducing the level of anxiety^(3,4,5,6,7)^.

It is important to highlight that, in addition to being a child’s right^(8)^, the use of play and playfulness has a therapeutic purpose, and is no longer a mere pastime activity, but can also affect children’s clinical condition and promote their recovery. Furthermore, it can strengthen bonds between professionals, children and their companions^(4,5,6)^. The incorporation of these technologies into health care ensures that care is comprehensive and humanized, centered on children and supportive of their autonomy^(3,5,6)^. For this reason, there is an urgent need for the use of these resources to be intentional and recurrent by professionals who are involved in providing care in the context of child hospitalization.

Currently, it is known that the nursing team incorporates the use of play and playfulness through playful languages, dolls, games and toys as mediators of interactions as well as using colorful uniforms and children’s paraphernalia^(4)^. It is important to highlight that, even with scientific evidence proving the importance of using play and playfulness^(4,6,9,10)^ in nursing professionals’ practices, their adoption is still incomplete and incipient^(4,7,10,11)^, and specific evidence for nursing technicians is scarce.

As a consequence of this fragility in the use of play, the establishment of interactions with children is limited as well as the construction of therapeutic bonds is difficult. This causes children to create a negative image of nursing technicians, reducing them to those who perform painful and unpleasant procedures^(4,7)^. For these reasons, it is essential that play be incorporated into the nursing work process and preside over the relationship between professionals and hospitalized children, ensuring its therapeutic potential^(4,7)^, promoting less traumatic hospitalization experiences.

In this regard, there is a gap in the literature that gives voice to nursing technicians, who, in the reality of Brazil, represent a professional category that is constantly in direct contact with hospitalized children. Thus, this article aims to look at the practices of nursing technicians working in a pediatric hospital unit. The question is: how do nursing technicians in pediatric hospital units perceive and adopt play and playfulness in their nursing care? The objective was to understand nursing technicians’ perceptions in a pediatric hospital unit regarding the use of play and playfulness in their professional practices.

## METHOD

### Study Design

This is an exploratory research with a qualitative approach, supported by the Symbolic Interactionism (SI) theoretical framework, focused on behaviors, meanings, senses, values ​​and beliefs^(12)^ related to play and playfulness in nursing technical practices with hospitalized children. SI understands that meanings are the result of social interactions. Individuals perceive the facts around them and assess and transform meanings about them, culminating in the emergence of actions and behaviors^(13)^. The interactional process is continuous and, therefore, the understanding and meaning of things is open. The guideline proposed by the COnsolidated criteria for REporting Qualitative research was adopted in this manuscript^(14)^.

### Place

This study was conducted in a city in the central-eastern countryside of the state of São Paulo, with an estimated population of 254,857 inhabitants, of which 16.97% were individuals aged 0 to 14 years^(15)^. The study setting was the pediatric inpatient unit of the city’s university hospital, which is one of the references for pediatric inpatient care in the region. The unit has 12 inpatient beds for children up to 12 years of age^(16)^.

### Population and Selection Criteria

At the time of this research, the nursing team consisted of 27 nursing technicians and 13 nurses, the latter divided between care and management. The entire nursing team’s working hours were 36 hours per week, divided into 12-hour shifts. In this regard, the inclusion criteria listed were to be a nursing technician working in the pediatric unit and to have worked there for at least one month, with time for social interaction and integration in the unit. Exclusion criterion was having been away from the unit for more than three months during the period in which the researcher was in the field.

All nursing technicians were invited to participate in the study. Two declined because, according to them, there were many studies being conducted at the hospital and, therefore, they did not see the need to participate in another one. The first author personally collected the data during the workday. The interview was scheduled via email. A total of 14 technicians returned the messages and agreed to participate in the interviews; two had left the institution; eight did not respond to attempts to contact them; and one responded that she was not available. The number of participants was based on a previously determined sample, but there was sufficient data, since concepts and their relationships in understanding the phenomenon under exploration were achieved.

### Data Collection

For data collection, two strategies were chosen: non-participant observation and semi-structured interview, all conducted by the first author. This article addresses the results obtained from interviews conducted with nursing technicians. This strategy was developed based on a set of previously formulated open questions. It is worth noting that, despite the existence of a script, this type of interview holds, when necessary, an opening for exploring participants’ thoughts, feelings and beliefs about the phenomena discussed^(17)^.

The interviews were conducted from March to December 2024, and took place on days and times agreed upon with participants. They took place both in person and virtually, with the latter being mediated by Google Meet^®^, according to interviewees’ availability. The questions to be used were discussed and reviewed in the research group to which the authors are affiliated, and were also submitted for analysis by pediatric nurses who work with the topic addressed in this study.

As an initial trigger, the following question was asked: how do you perceive your use of play and playfulness in your practices with children? Then, in an articulated manner with the above, the other questions were presented: how do you think this way of thinking/acting came about? What meaning do play and playfulness have for you as a nursing technician? How do play and playfulness relate to your professional practice? Thinking about the university hospital pediatrics unit, tell me what motivates you to use play and playfulness. Along the same lines, tell me what discourages you from using play and playfulness. If participants discussed the intention of one of the questions, it was not presented. Likewise, if they made statements that could be explored with other questions, it was presented.

The mean length of interviews was approximately 32 minutes. Both the in-person and virtual interviews were recorded in audio on an electronic device and transcribed in full using Transkriptor^®^ software. Afterwards, transcription was double-checked to validate the material. Transcriptions were not validated by participants.

### Data Analysis and Treatment

The analysis of data obtained occurred simultaneously with the interviews and was supported by the reflective thematic analysis proposed by Braun and Clarke^(18)^. This analytical process involved familiarization with the data, in which transcripts were read repeatedly to highlight the elements associated with the phenomenon under analysis. Afterwards, coding was carried out, in which transcripts were coded in light of the question and study objective. In the phase of searching for topics, the codes obtained in the previous phase were grouped into topics. In topic review, they were validated. Subsequently, the topics were defined and named, involving the detailed writing of the analysis of each one. Finally, the final draft was written with the integrated writing of analytical narrative as a whole, in conjunction with literature^(18)^. The entire analysis was conducted by the first author and then with the second author and, in a third moment, discussed with the others.

### Ethical Aspects

The study was submitted to and approved by the Research Ethics Committee, under Opinion 5,983,460 and Certificate of Presentation for Ethical Considerations 67333623.0.0000.5504. All ethical precepts were respected, in accordance with standards of Resolution 510 of 2016. It is worth highlighting that all scientific rigor was met, including the joint reading and signing of the Informed Consent Form. Participants’ identities were preserved, being identified with the name of a toy/game that they liked most in childhood, of their choice.

## RESULTS

Fourteen nursing technicians were interviewed, all female, working on four shifts, two day shifts and two night shifts. The minimum age was 33 years and the maximum was 58, with an average of approximately 46 years. Regarding the time since training as a nursing technician, the mean was 18 years, with a minimum of ten and a maximum of 28 years. Time working in pediatrics ranged from one year and four months to 21 years, while time working in the pediatrics listed in the study ranged from one year and four months to nine years.

Nursing technicians’ behaviors in relation to play and playfulness were supported by two meanings: (1) their adoption is in line with humanized practices; and (2) their adoption favors the execution of nursing procedures. These meanings were unfolded by professionals’ personal attributes, the outcomes experienced in the use of resources, the organizational context, and interactions in professional training as well as by the experience with children close to them.

The topics and subtopics obtained from data analysis (Chart 1) cover the scope of this study regarding nursing technicians’ perceptions in a pediatric hospital unit regarding the use of play and playfulness in their professional practices.

**Chart 1:** Coding of topics and subtopics derived from the analysis of data obtained in the study – São Carlos, SP, Brazil, 2024.

Topic	Subtopics
Purposes of adopting play and playfulness	Execution of procedures Humanization of practices
Determinants of the adoption of play and playfulness	Professional training Interactions with children Personal attributes Outcomes of use Organizational context

Source: the authors, 2024.

Topic: Purposes of Adopting Play and Playfulness

In this topic, participants listed two central purposes for the use of play and playfulness in their practices, namely “execution of procedures” and “humanization of practices”.

Subtopic: Execution of Procedures

The behavior of using play and playfulness was supported by nursing technicians due to the contributions they make to the execution of technical procedures, understood as the purpose of their profession. For them, the advantages were mainly related to the reduction of time to perform these procedures, due to the collaboration achieved with children.

*The biggest motivation for me is to speed up my process, the purpose I have when I enter the child’s room. The biggest advantage is the issue of optimized time demand, because it speeds up my service, I gain more time.* (Hopscotch)

*Sometimes people turn their profession into a mechanical one, as if it were a production line, and they think that doing this will take up their time, when in fact it will save them time, because the procedure will become easier and faster to do.* (Colored elephant)

Furthermore, it was reported that the use of play and playfulness favored the execution of procedures by strengthening relationships with children, promoting distraction and relaxation throughout the procedure, in addition to allowing information about the technique to be transmitted. With this, the safety and comfort of children were ensured, alleviating patients’ fears, including in relation to professionals.

*Playing helps children create a bond with the nursing team. When children strengthen their bond with professionals through play and playfulness, it becomes much easier to carry out routine care. Toys and games help children relax; they help them understand their process and suffer less from procedures.* (Dodgeball)

*I think that when you play with a child, you are becoming like them. So, they will look at you and think “Wow, they are a child just like me, how cool, I can trust them”. And this makes them learn to trust you and helps a lot.* (Jump rope)

*I realize that when we play some kind of game with children, make up a story, talk, something, outside of what they are going through, it takes the focus off that moment and they start to imagine something else. So, it is easier and less painful for them to go through that procedure, and it is easier for us too, because we can, especially during the puncture, have less chance of error, because children will move their arm less, you will prick them less often.* (Shuttlecock)

When exemplifying the contributions related to execution of procedures, they highlighted that there is no success when their execution is carried out in a mechanical way, since they scare and destabilize children, aspects that impact the development time of procedures.

*You have to be very careful: play, talk, be patient. Because sometimes things are hectic, we want to do things quickly and we can’t.* (Puzzle)

*I noticed that when we don’t use anything and just do things automatically, the child starts to cry, and then it takes longer for us to do things.* (Hopscotch)

Despite this, there were participants who acknowledged that they did not use play and playfulness in their practices, because they meant that they were being charged for carrying out procedures and not for using these resources. In this context, there were those who said that such activities should be directed to another professional, whose scope was to comply with the medical prescription. Furthermore, they understood that incorporating these playful attitudes would delay, overall, the completion of their tasks.

*I don’t usually use it because we really don’t have time for it. When I’m on duty, I’m responsible for more than one child, and there are several procedures. And I believe that it’s not ideal at this time to use play for the nursing technician. As I told you, I have my duties and I have to carry them out, I have to be able to handle them, because we have a medical prescription that we have to follow. And that takes up our work time. So, I’m not going to demand of myself something that I can’t handle. This has to be developed by another professional, not by the same professional who is providing care.* (Chanting)

*Since I’m still new to pediatrics, I don’t have the dexterity that other people have of doing things really quickly. So, sometimes, I think that if I stop for a minute to play, I’ll be late, and between playing and being late, I’d rather not play and do things right.* (Drawing)

Subtopic: Humanization of practices

For nursing technicians, playing, toys and playfulness were related to “being a child”, even in the context of hospitalization, being important in the relationship with patients from the moment of admission until discharge.

*For me, toys, as a technique, are something that saves, because they are very important for children. I think that a child without toys is not a child, and children have this, to laugh, to play.* (Puzzle)

*I have already understood that play, playfulness, is part of the process from children’s admission to treatment, until children leave, celebrating, playing, celebrating with them when they leave. I think this has to be part of pediatrics, because it is you entering their little world.* (Town, country, river)

For participants, recognizing children as people with rights led them to make an effort to interact with them, guaranteeing them a voice and a place in a comfortable relationship. In this sense, their behaviors were to intentionally listen to and talk to patients, through play and playfulness. In their conversations, they intended to provide children with an understanding of what was going to happen, especially when performing procedures.

*People say, “Oh, it’s just a child,” but I really like to explain the entire procedure I’m going to do in a way that they can understand.* (Drawing)

*Sometimes, depending on children’s size, I go down to talk to them, so they feel more comfortable talking.* (Town, country, river)

*It’s a way of understanding, of caring too, because if I say, “I’m going to stick a needle in”, children will have a fit. We also can’t say that it won’t hurt. We say that it’s going to give them a little prick and everything, that it’s a little ant, something so they don’t get too scared.* (Barbie)

Some nursing technicians recognized hospitalization as a traumatic and hostile event for children, as they were outside their family environment and under restricted control of the situation. In this scenario, play was conceived by them as a facilitator and mediator of more positive experiences.

*Children were placed in a hostile environment, with strangers manipulating them. They are already in an environment outside their home; they are already sick; they are already in a delicate moment.* (Doll)

*So, you try, in the experience, to remove that moment, to remove that tension, because everyone is outside their environment, their home, their little room. So, we try to alleviate it as much as possible so that children do not have so many traumas.* (Shuttlecock)

Given this fact, according to nursing techniques, playing and playfulness in their practices are related to promoting smiles and well-being of children, favoring their recovery.

*The biggest advantage of using play, I think, is more associated with patients, with the feeling it awakens in children. Children become happier; we see that they are happy.* (Hopscotch)

*What motivates me to use play and playfulness is children’s well-being. You see a smile on their face. For me, that has a meaning of love.* (Pogo stick)

*I see* (play and playfulness) *as positive for children. It helps a lot in their improvement. So, if they feel more welcomed, more loved, more important, they will get better faster.* (Playhouse)

Topic: Determinants of the adoption of play and playfulness

Subtopic: Professional training

According to participants’ reports, during the vocational course, they were told that the role of nursing technicians was to perform procedures and that they should not be involved with the person they were caring for. However, when they experienced the interactions in the pediatric unit, the meanings taught were relativized and began to mean that involvement with children and the use of play and playfulness are possibilities for their work.

*My academic training did not address this topic with me* (play and playfulness). *We simply enter a pediatrics ward and realize that everything is more colorful, everything is more drawn. So, intuitively we deduce that there should be more play and more use of play and toys there.* (Dodgeball)

*During my training, they said, “Don’t get involved”, but there is no way you can’t get involved. I think that, in order to provide good treatment there* (in the pediatric unit), *to provide good care, I think you have to get involved.* (Drawing)

*When I took the course* (nursing technician), *the teachers said, “You can’t create a bond with patients, you can’t talk like this, you can’t talk like that, you have to go in and do it”. And I realized that in practice that’s not how it works.* (Doll)

Subtopic: Interactions with children

Relationships with children, especially those experienced in the personal and family trajectory of each nursing technician, provoked internalized reflections about the importance of considering children, listening to them and welcoming them. In view of this, they value play and playfulness.

*Look, my life is surrounded by children. I have a daughter at home, so I treat children the same way I treat her here at home. I’ve always been very close to her, very close to my nieces, so I think the fact that my house is always full of children made it much easier for me to have contact with children here at the hospital.* (Drawing)

*At first, I was very scared, because I thought a lot about my daughter. So, I thought, “Wow, it must be really sad to have a child in this situation and everything”. And then, as a mother, I also kind of took it, like, “Oh, how would I like my daughter to be treated?” It was more or less like that, and then I treated her like that, and it just flowed.* (Barbie)

*I’ve always had a good relationship with children, I’ve always had a great ability, even before I started working in nursing. I really like the relationship with children. So, I already had this with me and then I realized that, as I applied it, my work became easier, and then I just kept improving, using it more and more and seeing that the results were good.* (Colored elephant)

Subtopic: Personal attributes

In this scenario, being cheerful, fun and playful were personal characteristics listed by participants, which they considered to be reflected in the use of play and playfulness in interactions with children. They reported feeling good about this way of being at work in pediatrics, and described that they are recreating presences, which brings satisfaction and increases their desire to maintain and invest in behavior.

*I’ve been a happy person since I can remember. I think this spirit has been inside me since I was born, to be happy, to make people happy, to always laugh and so on, and I really like children.* (Pogo stick)

*Oh, I use it all day long* (playing and being playful). *Every time I have contact, I play with them. I sing. If they’re crying, I pick them up, make fun of them, walk around the hospital with them showing them the plants in the garden, the toys. I do this all the time, all day long.* (Child’s play)

Subtopic: Outcomes of use

By adopting play and playfulness in their practices, nursing technicians reported that their behavior of use was fed back, once they constructed the meaning of being a demonstrator of an empathetic and affectionate act that qualifies their practice of care, reaching the person who was with children. Thus, use was symbolized as an attenuator of tension between technician, child and companion, promoting closer relationships and satisfaction for all.

*I see the improvement in children’s condition. I see that children’s improvement is immediate. That’s what motivates me. The more children react to play, the more I want to work on it with them. Playing and play provide this for children and the team; it’s good for both.* (Dodgeball)

*I’ll tell you this: there’s children’s side, which is difficult for them because they’re scared, because they’re in pain, they’re sad, and there’s our professional side, because we also have that tension. So, I think that using play and playfulness relieves both sides, both parties. I think it eases that moment for me and also for children.* (Chanting)

Subtopic: Organizational context

In participants’ perception, the institution did not encourage the use of play and playfulness by nursing technicians, in addition to not valuing the unit’s playful environment. They saw this as an outcome of the disease-centered approach and the concern with cost containment present in the institution. This led the participants to mobilize, on their own, strategies so that play and playfulness would be present in their practices and in their daily work.

*The institution does not encourage us, so much so that we are the ones who run after things* (for fun), *we are the ones who decorate here by buying everything with our own money. We take money out of our own pockets to make the environment a little better.* (Doll)

*I understand that nursing technicians can use fun and play, but here, in this institution, we do not have this encouragement.* [...] *I believe that it is more a problem of the institutional culture that focuses too much on the disease process, not so much on the human process of children, families.* (Dodgeball)

*There is no encouragement whatsoever from the institution, nor is there any provision of games or toys.* [...] *this has greatly discouraged* (the use of play/fun). (Playhouse)

In addition to the above, most of interviewees reported that playing, playfulness and the toy library were not topics addressed by the institution in terms of continuing education or guidance for their practices. They also commented that the relationship with technologies and the environment happened intuitively.

*Since I started working in pediatrics, I have never received any training or guidance related to playing, playful activities or the use of a toy library. I think it would be very important.* (Child’s play)

*Look, I’ve been here for a year and eight months, and I haven’t had any training to date. Here, the courses are more focused on techniques and procedures, but I haven’t seen this part of playfulness, playfulness, humanization.* (Jump rope)

*We have several training courses on the technique, but not on this issue that could make the technique easier. And I think that since many people don’t put this into practice, it would be worthwhile to do this type of incentive, because it’s not encouraged, it’s not listed as something important.* (Colored elephant)

Thus, the pediatric unit’s physical space, with the existence of a toy library/toys, was praised by the study participants, contributing to their adoption in their practices, if they wished to do so.

*I think the space there is great, it’s big. Sometimes we interact with the kids by playing ball there, in the middle of that hall, sometimes in the playroom.* (Pogo stick)

*You can use the toys and play equipment in the bedrooms, in the courtyard, which has that huge leisure area, and in the playroom too.* (Hopscotch)

In this scenario, the toy library was recognized by the participants as promoting the incorporation of the resources that are there into their professional actions, acting in children’s recovery and ensuring that the hospitalization experience is not so traumatic, even if its use was not encouraged by the institution.

*It’s a place* (the playroom) *that brings lightness to our work here. Sometimes I go there, do something, or take a child. I see that it brings lightness in a heavy environment, which is the hospital environment. It’s the only space where children remember that they are a child. So, I think the playroom plays a very important role in encouraging children.* (Doll)

*Technicians are not encouraged to use the playroom; the initiative is individual for each professional. Some have more initiative and others have less.* [...] *I believe that there should at least be training for the nursing team that will work in pediatrics or any place where children are present.* (Dodgeball)

## DISCUSSION

The meanings and actions of using play and playfulness emerge and are shaped by the interactional process developed between nursing technicians and hospitalized children, and are involved and influenced by the organizational context. Perspectives used to define play and playfulness as essential elements in the care of hospitalized children derive from professional and personal experiences and beliefs, internalized by the technicians, which relate them to humanization and reduction of possible traumas.

Hospitalization is considered a complex experience for children, with potential for stress and trauma, as it is for their companions. Both are exposed to an unknown environment and have their routines changed^(2,19)^. This fact was reported by most of participants in this study, who stated that playing emerges as a mediator of more positive experiences during children’s stay in the hospital. This demonstrates the need for institutions to invest in the pediatric unit’s ambiance, with the aim of making that environment more welcoming and comfortable for their patients^(1)^ and, consequently, promote and sustain the culture of playing in the unit.

It is currently known that playing and playfulness provide several benefits during a child’s hospitalization, including: minimizing feelings of sadness or bringing joy; aiding children’s recovery; facilitating interaction with professionals; minimizing pain and discomfort during the procedure; providing sensory stimulation; making children calmer and less stressed; facilitating acceptance of the procedure; fostering the creation of bonds; and strengthening trust in professionals^(11,20)^. All of the developments highlighted in the literature were also discussed by the participants in this study.

It is worth highlighting that playing is a child’s right and inherent to them^(21)^. It is also the language through which they express their needs. Therefore, promoting care that values ​​playfulness provides the joy of “being a child”. This supports participants’ view in this study that children, as individuals, need a different perspective from that of adults, with the ability to reveal perceptions and needs. For the nursing team, when professionals are willing to play, in addition to meeting children’s needs and promoting interaction, they are also at the same level of thought, constructing and reconstructing their meanings and promoting behavioral changes^(13)^, aiming at humanized care^(4)^. They understand that this egalitarian movement makes the situation interesting for children, who promptly transforms this opportunity into a possibility for shared play with professionals^(4,13)^. Furthermore, it allows children to express their feelings and emotions, in addition to better understanding the process and the need for hospitalization^(5,13)^.

According to this and other studies^(4,5,9)^, the use of play and playfulness also facilitates the performance of nursing procedures, especially in techniques such as puncture and/or administration of intravenous medications, since children are calmer and more relaxed, being able to cooperate more with professionals. In addition, such practices promote closer bonds, favor recovery and help relieve anxiety and tension caused by hospitalization^(5,9,22)^.

On the other hand, some of the participants interviewed stated that the use of play and playfulness is part of care practices of other professionals, and their role is to perform procedures and execute medical prescriptions. This attitude is in line with the results found in the literature, which point to the concept of play as an “extra” activity that is not part of nursing’s responsibility, and is not included in the list of its competencies^(7,9,23)^. Therefore, it is clear that play is not included in nursing, having a secondary and informal role, voluntary and non-institutionalized, which is not part of the nursing care process for children. Thus, the need for nursing techniques to re-signify the use of these resources is reinforced, causing attitudinal changes^(13)^, which encourage the assumption of play as inherent to nursing. The link between professionals and physicians, despite belonging to the nursing team, is noteworthy, an interesting aspect to be explored in future studies. Furthermore, there is a reflection on the fact that nursing prescriptions are not directed at play.

In any case, there were participants who demonstrated positive attitudes towards playing and playfulness. As demonstrated in other studies^(4,7,9,22)^, nursing technicians believe that these resources provide benefits during children’s hospitalization and, therefore, developed the perception that they are essential for the recovery, well-being and happiness of their patients^(24)^. Therefore, they support and defend their use^(11)^, seeking to sustain them, like those who bring resources to be able to use them in their professional work, since they do not have full institutional support for their incorporation. How are institutions with pediatric units considering play and playfulness in their cost agenda?

When discussing the factors that favor the use of play and playfulness in nursing technical practices, the existence of intrinsic and extrinsic factors for professionals was noted^(9)^. Intrinsic factors include promoting children’s recovery and helping them deal with the stress of hospitalization, which makes them feel motivated and satisfied. According to the interviewees, being happy, having fun and playing brought benefits not only to children, but also to themselves, which further encouraged the use of these practices, a result similar to that found in literature^(7,9,22,23)^. They also recognize that when children play with nurses, they (re)signify their image as someone more welcoming and playful^(7,13)^.

Extrinsic factors are associated with the presence of a toy library and other resources available there^(9)^. The hospital toy library helps children go through the hospitalization process by gaining confidence, facilitating adaptation and minimizing their suffering. It is a space where they can be closer to the routine they had outside the hospital. For the nursing team, it can help them be closer to children and their family, facilitating procedures and exams, making them less painful and contributing to adherence to treatment, which can increase children’s chances of recovery^(25)^. In this study, it was evident that the existence of a large space in pediatrics symbolized a motivator for the use of play and playfulness. However, the lack of incentive from the institution to explore the environment led to a discouraging factor for involvement in playful activities. Therefore, it is evident that institutions need to redefine the belonging and inclusion of play, games and the toy library in the nursing team’s professional work, with effective support and recognition of this type of intervention^(13)^.

Another factor that acted as a demotivator in the present study was the lack of institutional guidance and guidance, especially regarding the provision of professional training that addressed play and playfulness with children, which would make them more confident and secure to work in pediatrics. Added to this are the gaps faced during the vocational nursing course, which advised them not to establish bonds with their patients. This aspect requires renewal and is indicated as the subject of future studies.

Other research carried out revealed that the demand for work, the routine of procedures, the mechanical nature of care, lack of time, the scarcity of infrastructure and ongoing training were identified as determining factors for the low viability of its use by the nursing team, with questions about the place of play in institutional culture^(7,11,26)^. Furthermore, another study carried out^(4)^ also reinforced these findings, revealing the non-incorporation of play into the nursing process in institutions, reinforcing the non-recognition of the play-care dyad, an advance that needs to become part of the health care agenda, especially in the pediatric setting.

It is also important to emphasize the importance of nurses’ role in the attitude of their team. They must be trained to develop play and playfulness in an intentional, planned and integrated way into the care plan, in addition to encouraging and maintaining their team trained for care, leading them to change the meanings attributed, until then, to these technologies, which, when experienced in social interactions, provoke changes in behavior^(7,13)^. In our study, it was demonstrated that the presence of a nurse who encourages their team makes them feel more willing and motivated to include these resources in their practices.

Therefore, it is clear that hospital authorities and managers need to be aware of the importance of play and playfulness for hospitalized children, with the aim of providing their full support to the implementation of playfulness and providing the necessary resources to facilitate the integration and use of play as a strategy to promote comfort, alleviate anxiety and fears, allow communication and the expression of fears, and promote the recovery and general well-being of hospitalized children^(9)^. This institutional care creates a culture and promotes, in social interactions^(13)^, the sharing of the uniqueness of resources for humanization and meeting needs, such as playing for children.

This research, by giving voice to nursing technicians working in pediatric units, seeks to raise awareness among professionals and managers of these institutions about the importance and benefits of using play and playfulness, with the possibility of provoking changes and redefinitions^(13)^ that promote the humanization of pediatric care, with investments in this professional category. The limitations of this study are the fact that it was developed in a single hospital setting, with a significant number of participants not being included in the second stage of the research. Furthermore, the fact that the interviews took place in person and online can also act as a limiting factor, since different mediations, explorations and presences occur. However, since it was the participant’s choice, it is believed that they chose the form with which they felt most comfortable, which favored the narratives.

The SI framework was powerful in focusing on participants’ behaviors, highlighting the meanings that structured them as well as the elements of the social scene that mobilized processes in the self. Interactions with children stood out in this context, and the process of developing an awareness of “oneself” as a nursing technician also deserves to be highlighted, based on a process of reflexivity that “inquires” about what is “socially” and institutionally placed on the profession. This process makes explicit the emergence of the “me” acting on the awareness of the self, with contributions to decision-making about the incorporation of play and playfulness.

## CONCLUSION

This study allowed us to understand the perceptions of nursing technicians working in a pediatric inpatient unit regarding the use of play and playfulness in their professional practices. The prevalence of the symbolism of these resources contributing to the execution of technical procedures and the formation of bonds was observed. However, they pointed out a context that was not very supportive of this use and that led to the meaning of these resources being the scope of other professionals. There were participants who recognized and understood the resources as aligned with humanized care, with efforts to invest in and maintain behaviors that incorporate them.

Thus, it is concluded that there is a need to invest in the discussion about the importance of investing in nursing technicians as a category that can contribute to and promote play and playfulness during hospitalization. To this end, institutional positions need to be renewed urgently, with emphasis on those involved in the training of these professionals, their employers and the professional council. Nursing technicians can expand and sustain the presence of play and playfulness in the daily routine of children’s hospitalization. Play and playfulness are therapeutic instruments in this scenario; providing infrastructure and support for their widespread use is essential; and professional and continuing education needs to recognize and address the topic within what is pertinent to each profession. In any case, a good start is for nursing technical training centers to address this topic, developing skills and competencies for use within their professional scope, with contributions to the quality of care offered in hospital pediatric units.

## Data Availability

The dataset supporting the results of this study is not publicly available.
