# ERRATUM: Nursing technicians’ perceptions about the use of play and playfulness in professional practices

**DOI:** 10.1590/1980-220X-REEUSP-2025-0024eren

**Published:** 2025-08-11

In the article “Nursing technicians’ perceptions about the use of play and playfulness in professional practices”, with DOI code number: https://doi.org/10.1590/1980-220X-REEUSP-2025-0024en, published in the journal Revista da Escola de Enfermagem da USP, 59:e20250024, 2025, in the page 1:

**Where it was written:**

Gabriele Petruccelli^1^

Monika Wernet^2^

Aline Oliveira Silveira^3^

Edmara Bazoni Soares Maia^2^

Cristiane Leite de Almeida^4^

Fernanda Manuela Loureiro^5^

^1^ Universidade Federal de São Carlos, Programa de Pós-Graduação em Enfermagem, São Carlos, SP, Brazil.

^2^ Universidade Federal de São Carlos, Departamento de Enfermagem, São Carlos, SP, Brazil.

^3^ Universidade de Brasília, Departamento de Enfermagem, Brasília, DF, Brazil.

^4^ Escola Técnica Estadual Paulino Botelho, São Carlos, SP, Brazil.

^5^ Universidade Católica Portuguesa, Lisboa, Portugal.

**Should read:**

Gabriele Petruccelli^1^

Monika Wernet^2^

Aline Oliveira Silveira^3^

Edmara Bazoni Soares Maia^4^

Cristiane Leite de Almeida^5^

Fernanda Manuela Loureiro^6^

^1^ Universidade Federal de São Carlos, Programa de Pós-Graduação em Enfermagem, São Carlos, SP, Brazil.

^2^ Universidade Federal de São Carlos, Departamento de Enfermagem, São Carlos, SP, Brazil.

^3^ Universidade de Brasília, Departamento de Enfermagem, Brasília, DF, Brazil.

^4^ Universidade Federal de São Paulo, Escola Paulista de Enfermagem, São Paulo, SP, Brazil.

^5^ Escola Técnica Estadual Paulino Botelho, São Carlos, SP, Brazil.

^6^ Universidade Católica Portuguesa, Lisboa, Portugal.

