# Supplementary material for: Nursing technicians’ perceptions about the use of play and playfulness in professional practices
Source: Rev Esc Enferm USP. 2025 Jul 14;59:e20250024. doi: 10.1590/1980-220X-REEUSP-2025-0024en (PMC12274076; doi:10.1590/1980-220X-REEUSP-2025-0024en)
Supplement: Translation

# Translation: Percepções de técnicas de enfermagem acerca do uso do lúdico e do brincar nas práticas profissionais

**DOI:** 10.1590/1980-220X-REEUSP-2025-0024pt

**Authors:** Gabriele Petruccelli, Monika Wernet, Aline Oliveira Silveira, Edmara Bazoni Soares Maia, Cristiane Leite de Almeida, Fernanda Manuela Loureiro

**Affiliations:** 1 Universidade Federal de São Carlos, Programa de Pós-Graduação em Enfermagem, São Carlos, SP, Brasil.; 2 Universidade Federal de São Carlos, Departamento de Enfermagem, São Carlos, SP, Brasil.; 3 Universidade de Brasília, Departamento de Enfermagem, Brasília, DF, Brasil.; 4 Universidade Federal de São Paulo, Escola Paulista de Enfermagem, São Paulo, SP, Brasil.; 5 Escola Técnica Estadual Paulino Botelho, São Carlos, SP, Brasil.; 6 Universidade Católica Portuguesa, Lisboa, Portugal.

**Keywords:** Enfermagem Pediátrica, Hospitalização, Criança, Brincadeiras e Brinquedos, Técnicos de Enfermagem

## Abstract

**Objetivo:**

Conhecer as percepções de técnicas de enfermagem de uma unidade de internação pediátrica acerca do uso do lúdico e do brincar em suas práticas profissionais.

**Método:**

Estudo exploratório de abordagem qualitativa, apoiado no referencial teórico do Interacionismo Simbólico, desenvolvido em unidade pediátrica de um hospital universitário do interior paulista. A coleta de dados aconteceu no período de março a dezembro de 2024, por meio de entrevistas semiestruturadas, contando com 14 participantes. A análise temática reflexiva sustentou a apreciação dos dados.

**Resultados:**

Os temas “Finalidades da adoção do brincar e do lúdico” e “Determinantes da adoção do brincar e do lúdico” revelaram que as técnicas de enfermagem vinculam os recursos com a execução de procedimentos técnicos e formação de vínculo. Porém, destacaram um contexto pouco sustentador desse uso e indutor do significado de serem esses recursos de escopo de outros profissionais.

**Conclusão:**

Predominou o uso do brincar e do lúdico para a execução de procedimentos, com lugar secundário, informal, voluntário e não institucionalizado no cuidado de enfermagem à criança.

## INTRODUÇÃO

A hospitalização representa para a criança um evento complexo gerador de estresse, ansiedade, sofrimento, angústia e tensão, com potencial para se transformar em uma experiência traumática. Além disso, tanto a criança quanto aquela pessoa que a acompanha ficam expostas ao desconhecido por terem o seu cotidiano modificado^(1,2)^.

Ainda, durante o período de internação, ela pode vir a ser exposta a procedimentos diagnósticos e terapêuticos de potencial álgico, assim como ficar afastada do ambiente familiar, dos amigos e do espaço de educação^(1,3)^.

Nesse cenário, é usual enfrentar restrições ao brincar, uma ocupação essencial^(1,3)^. A incorporação do brincar e do lúdico está assinalada como transformadora do ambiente hospitalar, sobretudo por simbolizar o familiar à criança^(3,4)^ e trazer um sentido de normalidade e continuidade de sua vida. Isso faz com que esses recursos atuem como uma válvula de escape, auxiliando no desenvolvimento de estratégias para enfrentar a doença e a internação, favorecendo distrações e divertimentos, facilitando a assimilação de informações, e diminuindo o nível de ansiedade^(3,4,5,6,7)^.

Torna-se necessário destacar que, além de ser um direito da criança^(8)^, o uso do lúdico e do brincar apresenta cunho terapêutico, deixando de ser uma atividade meramente de passatempo, sendo capaz de atuar na condição clínica da criança e promover sua recuperação. Ainda, possibilita o fortalecimento de vínculos entre o profissional, a criança e seu acompanhante^(4,5,6)^. A incorporação dessas tecnologias no cuidado em saúde favorece que a assistência seja integral e humanizada, centrada na criança e sustentadora de sua autonomia^(3,5,6)^. Por esse motivo, há premência que o uso desses recursos aconteça de forma intencional e recorrente por profissionais que estejam envolvidos em sua assistência no contexto da hospitalização infantil.

Atualmente, sabe-se que a equipe de enfermagem incorpora o uso do brincar e do lúdico a partir de linguagens lúdicas, bonecos, brincadeiras e brinquedos como mediadores das interações, assim como fazem uso de uniformes coloridos e apetrechos infantis^(4)^. É importante destacar que, mesmo com as evidências científicas comprovando a importância do uso do lúdico e do brincar^(4,6,9,10)^ nas práticas de profissionais de enfermagem, sua adoção ainda é lacunar e incipiente^(4,7,10,11)^, e as evidências específicas aos técnicos de enfermagem são escassas.

Como consequências dessa fragilidade no uso do brincar, o estabelecimento de interações com a criança fica limitado, assim como a construção de vínculos terapêuticos é dificultada. Isso faz com que ela crie uma imagem negativa do técnico de enfermagem, reduzindo-o ao executor de procedimentos dolorosos e desagradáveis^(4,7)^. Por esses motivos, torna-se imprescindível que esse recurso seja incorporado ao processo de trabalho de enfermagem e presida a relação entre o profissional e a criança internada, fazendo com que seja assegurado seu potencial terapêutico^(4,7)^ e promovendo experiências menos traumáticas de hospitalização.

Nesse sentido, percebe-se a existência de uma lacuna na literatura ao dar voz para os técnicos de enfermagem, que, na realidade do Brasil, representam uma categoria profissional constante na relação direta com a criança hospitalizada. Dessa forma, o presente artigo se propõe a olhar para as práticas de técnicas de enfermagem atuantes em uma unidade pediátrica hospitalar. Questiona-se: como técnicas de enfermagem de unidades pediátricas hospitalares percebem e adotam o lúdico e o brincar na sua assistência de enfermagem? O objetivo foi de conhecer as percepções de técnicas de enfermagem de uma unidade de internação pediátrica acerca do uso do lúdico e do brincar em suas práticas profissionais.

## MÉTODO

### Tipo de Estudo

Trata-se de pesquisa exploratória, de abordagem qualitativa, apoiada no referencial teórico do Interacionismo Simbólico (IS), focada nos comportamentos, significados, sentidos, valores e crenças^(12)^ relativos ao lúdico e ao brincar nas práticas de técnicas de enfermagem junto à criança hospitalizada. O IS entende que as significações são resultantes de interações sociais. Os indivíduos percebem os fatos à sua volta e aferem e transformam significados acerca deles, culminando no surgimento de ações e comportamentos^(13)^. O processo interacional é contínuo e, por isso, o entendimento e sentido das coisas está aberto. A diretriz proposta pelo *COnsolidated criteria for REporting Qualitative research* foi adotada neste manuscrito^(14)^.

### Local

Estudo desenvolvido em um município do centro-leste do interior do estado de São Paulo com uma população estimada de 254.857 habitantes, sendo 16,97% representados por indivíduos de 0 a 14 anos^(15)^. O cenário da pesquisa foi a unidade de internação pediátrica do hospital universitário da cidade, o qual é uma das referências em internação pediátrica da região. A unidade conta com 12 leitos de internação para crianças com até 12 anos incompletos^(16)^.

### População e Critérios de Seleção

No momento desta pesquisa, a equipe de enfermagem era composta por 27 técnicas de enfermagem e 13 enfermeiras, essas últimas divididas entre assistenciais e gerenciais. A jornada de trabalho de toda a equipe da enfermagem era de 36 horas semanais, divididas em plantões de 12 horas. Nesse sentido, os critérios de inclusão elencados foram ser técnica de enfermagem atuante na unidade pediátrica e deter minimamente um mês de atuação na mesma, tempo para convívio e inserção na unidade. Já o critério de exclusão foi haver ocorrência de afastamento da unidade por tempo maior que três meses, durante o período no qual a pesquisadora esteve em campo.

Foram convidadas para participar da pesquisa a totalidade das técnicas de enfermagem. Duas não aceitaram, pois, de acordo com elas, eram desenvolvidas muitas pesquisas no hospital e, por isso, não viam necessidade em participar de mais uma. A captação foi realizada pela primeira autora, pessoalmente, durante a jornada de trabalho. Já o agendamento da entrevista aconteceu por meio de mensagem eletrônica. Um total de 14 técnicas retornaram as mensagens e aceitaram participar das entrevistas; duas haviam deixado a instituição; oito não responderam às tentativas de contato; e uma contou que não tinha disponibilidade. O número de participantes partiu de uma amostra previamente dada, porém houve suficiência de dados, já que foram alcançados os conceitos e suas relações na compreensão do fenômeno colocado em exploração.

### Coleta de Dados

Para coleta de dados, foram elencadas duas estratégias, como a observação não participante e a entrevista semiestruturada, todas conduzidas pela primeira autora. Este artigo aborda os resultados obtidos com as entrevistas realizadas junto às técnicas de enfermagem. Essa estratégia foi desenvolvida a partir de um conjunto de questões abertas previamente formuladas. Cabe destacar que, apesar da existência de um roteiro, esse tipo de entrevista detém, na necessidade, uma abertura para exploração de pensamentos, sentimentos e crenças dos participantes acerca dos fenômenos postos em diálogo^(17)^.

As entrevistas foram realizadas durante o período de março a dezembro de 2024, e aconteceram em dias e horários pactuados com as participantes. Elas ocorreram tanto presencialmente quanto virtualmente, sendo essas mediadas pela plataforma *Google Meet* ^®^, de acordo com a disponibilidade das técnicas. As questões a serem utilizadas foram discutidas e revistas no grupo de pesquisa ao qual as autoras estão inseridas, assim como foram submetidas à análise por enfermeiras pediátricas que trabalham com a temática abordada no presente estudo.

Como disparador inicial, era feita a seguinte colocação: como você percebe o seu uso do brincar e do lúdico nas suas práticas junto à criança? A seguir, de forma articulada ao exposto, apresentavam-se as outras perguntas: como acredita que surgiu essa forma de pensar/agir? Qual o significado que o brincar e o lúdico têm para você enquanto técnica de enfermagem? Como o brincar e o lúdico se relacionam com a sua prática profissional? Pensando na unidade de pediatria do hospital universitário, conte-me o que te motiva a utilizar o brincar e o lúdico. Nessa mesma linha, conte-me o que te desmotiva a utilizar o brincar e o lúdico. Se a participante discutisse a intenção de uma das perguntas, ela não era apresentada. Da mesma forma, se ela fizesse afirmações que pudessem ser exploradas com outras questões, ela era apresentada.

A duração média das entrevistas foi de aproximadamente 32 minutos. Tanto as pessoais quanto as virtuais foram gravadas em áudio por aparelho eletrônico e transcritas na íntegra com auxílio do *software Transkriptor* ^®^. Após, era realizada dupla conferência da transcrição para que ocorresse a validação do material. As transcrições não foram validadas pelas participantes.

### Análise e Tratamento dos Dados

A análise dos dados obtidos ocorreu concomitantemente às entrevistas e esteve apoiada na análise temática refletiva proposta por Braun e Clarke^(18)^. Esse processo analítico envolveu a familiarização com os dados, em que as transcrições sofreram leituras reiteradas destacando os elementos associados ao fenômeno posto em análise. Após, foi realizada a codificação, na qual as transcrições foram codificadas à luz da pergunta e do objetivo do estudo. Na fase de busca por temas, os códigos obtidos na fase anterior foram agrupados em temas. Já na revisão de temas, foi realizada a validação desses. Em seguida, foi feita a definição e nomeação dos temas envolvendo a escrita detalhada da análise de cada um. Por fim, realizou-se a redação final com a escrita integrada do conjunto da narrativa analítica de modo articulado com a literatura^(18)^. Toda a análise foi conduzida pela primeira autora e, em seguida, com a segunda autora e, em um terceiro momento, discutida com as demais.

### Aspectos Éticos

O estudo foi submetido e aprovado pelo Comitê de Ética em Pesquisa com Seres Humanos, sob Parecer nº 5.983.460 e Certificado de Apresentação para Apreciação Ética nº 67333623.0.0000.5504. Todos os preceitos éticos foram respeitados atendendo às normas da Resolução nº 510 de 2016. Vale a pena destacar que todo o rigor científico foi cumprido, incluindo a leitura conjunta e assinatura do Termo de Consentimento Livre e Esclarecido. As participantes tiveram suas identidades preservadas, sendo identificadas com o nome de um brinquedo/brincadeira que mais gostavam na infância, de sua escolha.

## RESULTADOS

Foram entrevistadas 14 técnicas de enfermagem, todas do sexo feminino, pertencentes aos quatro plantões, sendo dois diurnos e dois noturnos. A idade mínima foi de 33 anos, e a máxima, de 58, com uma média de aproximadamente 46 anos. Quanto ao tempo de formação como técnica de enfermagem, a média foi de 18 anos, com mínimo de dez e máximo de 28 anos. O tempo trabalhando na pediatria variou de um ano e quatro meses a 21 anos, enquanto o tempo trabalhando na pediatria elencada ao estudo variou de um ano e quatro meses a nove anos.

Os comportamentos das técnicas de enfermagem em relação ao lúdico e ao brincar estiveram sustentados por dois significados principais: (1) sua adoção alinha-se com as práticas humanizadas; e (2) sua adoção favorece a execução de procedimentos de enfermagem. Tais sentidos foram desdobrados pelos atributos pessoais das profissionais, pelos desfechos experienciados diante do uso dos recursos, do contexto organizacional, e das interações na formação profissional, assim como pela vivência com crianças próximas.

Os temas e subtemas obtidos com a análise dos dados (Quadro 1) contemplam os alcances do presente estudo quanto às percepções de técnicas de enfermagem de uma unidade pediátrica hospitalar acerca do uso do lúdico e do brincar em suas práticas profissionais.

**Quadro 1:** Codificação dos temas e subtemas derivados da análise dos dados obtidos no estudo – São Carlos, SP, Brasil, 2024.

Tema	Subtemas
Finalidades da adoção do brincar e do lúdico	Execução de procedimentos Humanização das práticas
Determinantes da adoção do brincar e do lúdico	Formação professional Interações com crianças Atributos pessoais Desfechos diante do uso Contexto organizacional

Fonte: autoria própria, 2024.

Tema: Finalidades da adoção do brincar e do lúdico

Subtema: Execução de procedimentos

O comportamento de uso do lúdico e do brincar esteve sustentado pelas técnicas de enfermagem por conta das contribuições que trazem para a execução de procedimentos técnicos, entendidos como a finalidade de sua profissão. Para elas, as vantagens relacionavam-se, majoritariamente, à redução do tempo para execução desses, devido à colaboração alcançada junto à criança.

*A maior motivação, para mim, é acelerar mesmo o meu processo, a finalidade que eu tenho ao entrar no quarto da criança. A maior vantagem é a questão da demanda do tempo otimizada, porque agiliza o meu atendimento, eu ganho mais tempo.* (Amarelinha)

*Às vezes, a pessoa torna a profissão mecanicista, como se fosse uma linha de produção, e a pessoa acha que fazer isso vai tomar o tempo dela, quando na verdade isso vai economizar o tempo dela, porque o procedimento vai se tornar mais fácil e mais rápido de fazer.* (Elefantinho colorido)

Ainda, foi relatado que o uso do brincar e do lúdico favorecia a execução de procedimentos por estreitar as relações com a criança, promovendo distração e relaxamento ao longo dele, além de permitir que as informações sobre a técnica fossem transmitidas mais facilmente. Com isso, a segurança e o conforto da criança eram assegurados, atenuando medos do paciente, inclusive em relação ao profissional.

*O brincar auxilia a criança a criar vínculo com a equipe de enfermagem. Quando a criança fortalece o vínculo com o profissional através do lúdico e do brincar, fica muito mais fácil a gente conseguir fazer a rotina dos cuidados. O brinquedo e o lúdico auxiliam bastante a criança a relaxar; eles ajudam ela a compreender o seu processo, a sofrer menos com os procedimentos.* (Queimada)

*Eu penso que quando você brinca com uma criança, você está se igualando a ela. Então, ela vai olhar para você e pensar: “Puxa, ela é criança que nem eu, que legal, eu posso confiar nela”. E isso faz ela aprender a confiar em você e ajuda muito.* (Pular corda)

*Eu percebo que quando a gente faz algum tipo de brincadeira com a criança, inventa uma historinha, conversa, fala, alguma coisa, fora daquilo que ela está passando, tira o foco daquele momento e ela começa a imaginar uma outra coisa. Então, é mais fácil e menos dolorido dela passar por aquele procedimento, e fica mais fácil para a gente também, porque a gente consegue, principalmente, na punção, ter menos probabilidade de erro, porque a criança vai mexer menos o braço, você vai furar menos vezes ela.* (Peteca)

Ao exemplificarem as contribuições relacionadas à execução dos procedimentos, ressaltaram que não há êxito quando sua execução é realizada de forma mecanizada, uma vez que isso assusta e desestabiliza a criança, aspectos que impactam o tempo de desenvolvimento dos procedimentos.

*Você tem que ter todo aquele cuidado: brincar, falar, ter paciência. Porque às vezes está corrido, a gente quer fazer rápido os procedimentos e não dá.* (Quebra-cabeça)

*Eu percebi que, quando a gente não usa nada e vai no automático mesmo fazer os procedimentos, a criança começa a chorar, e aí demora mais tempo para a gente fazer as coisas.* (Amarelinha)

Apesar disso, houve participantes que reconheceram não utilizar o lúdico e o brincar em suas práticas, por significarem que são cobradas pela execução dos procedimentos e não pelo uso desses recursos. Nesse contexto, houve quem significou que tais atividades deveriam ser direcionadas a outro profissional, sendo de seu escopo o cumprimento da prescrição médica. Ademais, entenderam que a incorporação dessas atitudes lúdicas atrasaria, no conjunto, a finalização de seus afazeres.

*Eu não costumo usar porque realmente a gente não tem tempo para isso. Quando eu recebo o plantão, eu sou responsável por mais de uma criança, e tem vários procedimentos. E eu acredito que não é o ideal nesse momento usar o lúdico para o técnico de enfermagem. Como eu te falei, eu tenho as minhas atribuições e eu tenho que realizar, tenho que dar conta, porque a gente tem uma prescrição médica que a gente tem que fazer. E aquilo ocupa o nosso período de trabalho. Então, eu não vou me cobrar aquilo que eu não vou dar conta de fazer. Isso tem que ser desenvolvido por um outro profissional, não pelo mesmo profissional que está na assistência.* (Cantarolar)

*Como eu ainda sou nova na pediatria, eu não tenho aquela destreza que o pessoal tem de fazer as coisas muito rápido. Então, às vezes, eu penso que, se eu parar um minuto para brincar, eu vou me atrasar, e entre brincar e me atrasar, eu prefiro não brincar e fazer as coisas certinhas.* (Desenhar)

Subtema: Humanização das práticas

Para as técnicas de enfermagem, o brincar, o brinquedo e o lúdico estiveram remetidos ao “ser criança”, inclusive quando no contexto de hospitalização, sendo importante na relação com o paciente desde o momento da admissão até a alta.

*O brinquedo, para mim, como técnica, é algo que salva, porque, para a criança, é muito importante. Eu acho que uma criança sem brinquedo, ela não é criança, e criança tem isso, de rir, de brincar.* (Quebra-cabeça)

*Eu já entendi que a brincadeira, o lúdico, ele faz parte desde a admissão da criança até o tratamento, até essa criança ir embora, comemorar, brincar, festejar com ela a saída dela. Eu acho que isso tem que ter na pediatria, porque é você entrar no mundinho deles.* (*Stop*)

Para as participantes, o reconhecimento da criança enquanto pessoa com direitos determinou que se esforçassem para interagir com ela, garantindo-lhe voz e vez sob um conforto relacional. Nessa direção, os comportamentos foram de, intencionalmente, escutar e conversar com o paciente, sob mediação do lúdico e do brincar. Nas conversas, intencionaram prover entendimento à criança do que iria ocorrer, sobretudo quando da realização de procedimentos.

*O pessoal fala assim: “Ah, é só uma criança”, mas eu gosto muito de explicar todo o procedimento que eu vou fazer de uma forma que ela entenda.* (Desenhar)

*Às vezes, dependendo do tamanho da criança, eu desço até ela para conversar, para ela se sentir mais à vontade na sua fala. (Stop)*

*É uma forma de compreensão, de carinho também, porque se eu falar: “Vou enfiar uma agulha”, a criança vai ter um treco. A gente também não pode falar que não vai doer. A gente fala que vai dar uma picadinha e tudo, fala que é uma formiguinha, algo para ela não assustar tanto.* (Barbie)

Parte das técnicas de enfermagem reconheceu a hospitalização como um evento traumático e hostil à criança, por estar fora de seu ambiente familiar e sob controle restrito da situação. Nesse cenário, o brincar foi concebido por elas enquanto um facilitador e mediador de experiências mais positivas.

*A criança foi colocada em um ambiente hostil, com pessoas estranhas manipulando ela. Ela já está num ambiente fora da casa dela; já está doente; já está num momento delicado.* (Boneca)

*Então você procura, na vivência, tirar aquele momento, tirar aquela tensão, porque todos estão fora do seu ambiente, da sua casa, do ser quartinho. Então, a gente tenta amenizar o máximo possível para a criança não ficar com tantos traumas.* (Peteca)

Diante desse fato, de acordo com as técnicas de enfermagem, o brincar e o lúdico em suas práticas se relacionaram à promoção de sorrisos e do bem-estar da criança, favorecendo sua recuperação.

*A maior vantagem de usar o brincar, eu acho que está mais associada ao paciente, ao sentimento que desperta na criança. A criança fica mais feliz, a gente vê que ela fica alegre.* (Amarelinha)

*O que me motiva a usar o lúdico e o brincar é o bem-estar das crianças. Você ver um sorriso nelas. Para mim, isso tem um significado de amor.* (Pula-pula)

*Eu vejo* (o brincar e o lúdico) *como positivos para a criança. Ajuda muito na melhora dela. Então, se ela se sente mais bem acolhida, mais amada, mais importante, ela vai melhorar mais rápido.* (Casinha)

Tema: Determinantes da adoção do brincar e do lúdico

Subtema: Formação profissional

De acordo com os relatos das participantes, durante a realização do curso técnico, receberam indicativas de ser o papel do técnico de enfermagem executar procedimentos e que não deveriam se envolver com a pessoa por elas cuidada. Porém, ao vivenciarem as interações na unidade pediátrica, as significações ensinadas foram relativizadas e passaram a significar que o envolvimento com a criança e o uso do brincar e do lúdico são possibilidades para o seu trabalho.

*A minha formação acadêmica não abordou esse tema comigo* (brincar e lúdico)*. A gente simplesmente entra numa pediatria e percebe que tudo é mais colorido, tudo é mais desenhado. Então, intuitivamente nós deduzimos que ali deva haver mais brincadeiras e haver mais o uso do lúdico e do brinquedo.* (Queimada)

*Durante minha formação, eles falavam: “Não se envolve”, mas não tem como você não se envolver. Eu acho que, para prestar um bom tratamento ali* (na unidade pediátrica), *prestar uma boa assistência, eu acho que você tem que se envolver.* (Desenhar)

*Quando eu fiz o curso* (técnico em enfermagem)*, as professoras falavam: “Você não pode criar vínculo com o paciente, você não pode falar assim, você não pode falar assado, você tem que chegar e fazer”. E eu percebi que na prática não é assim que funciona.* (Boneca)

Subtema: Interações com crianças

As relações com crianças, em especial aquelas vividas na trajetória pessoal e familiar de cada técnica de enfermagem, provocaram reflexões internalizadas acerca da importância de considerar a criança, escutá-la e acolhê-la. Diante disso, valorizam o brincar e o lúdico.

*Olha, a minha vida é cercada por crianças. Eu tenho uma filha em casa, então eu trato as crianças como eu trato ela aqui em casa. Eu sempre fui muito próxima dela, muito próxima das minhas sobrinhas, então eu acho que o fato de eu estar com a minha casa sempre cheia de crianças facilitou muito o meu contato com as crianças aqui do hospital.* (Desenhar)

*No início, eu me assustava muito, porque eu pensava muito na minha filha. Então, eu pensava: “Nossa, deve ser muito triste ter um filho nessa situação e tudo o mais”. E aí, como mãe, também eu fui meio que levando, tipo assim: “Ah, como é que eu gostaria que tratasse a minha filha?”. Foi mais ou menos essa pegada, e aí eu fui tratando assim, e simplesmente foi fluindo.* (Barbie)

*Eu sempre tive um bom relacionamento com criança, sempre tive muita facilidade, mesmo antes de começar a trabalhar na enfermagem. Gosto muito do relacionamento com a criança. Então, eu já trazia isso comigo e aí eu percebi que, conforme eu ia aplicando isso, o meu trabalho se tornou mais fácil, e aí eu só fui aprimorando, utilizando cada vez mais e vendo que o resultado era bom.* (Elefantinho colorido)

Subtema: Atributos pessoais

Nesse cenário, ser alegre, divertida e brincante foram características pessoais listadas pelas participantes, as quais elas consideravam reverberar no uso do brincar e do lúdico nas interações com a criança. Relataram que se sentiam bem com esse jeito de ser no trabalho na pediatria, e descreveram que recriam suas presenças, o que traz satisfação e incrementa seu desejo de manter e investir no comportamento.

*Eu sou uma pessoa alegre desde quando eu me conheço por gente. Eu acho que esse espírito está dentro de mim desde quando eu nasci, de alegre, fazer as pessoas felizes, sempre dar risada e tal, e eu gosto muito de criança.* (Pula-pula)

*Ah, eu uso o dia inteiro* (o brincar e o lúdico). *Toda vez que eu tenho contato, eu brinco com elas. Eu canto. Se elas estão chorando, eu pego elas no colo, faço graça, ando com elas pelo hospital mostrando as plantinhas do jardim, os brinquedos. Eu faço isso sempre, o dia inteiro.* (Amiguinha)

Subtema: Desfechos diante do uso

Ao adotar o brincar e o lúdico em suas práticas, as técnicas de enfermagem relataram ter seu comportamento de uso retroalimentado, uma vez construído o significado de ser demonstrador de um ato empático e afetuoso que qualifica sua prática de cuidado, alcançando a pessoa que estava com a criança. Assim, o uso foi simbolizado como um atenuador da tensão entre técnico, criança e acompanhante, promotor de estreitamento relacional e de satisfação a todos.

*Eu vejo a melhora do estado da criança. Eu vejo que a melhora da criança é imediata. É isso que me motiva. Quanto mais a criança reage ao lúdico, mais eu tenho vontade de trabalhar aquilo com ela. O brincar e o lúdico proporcionam isso para a criança e para a equipe; fica bom para ambos.* (Queimada)

*Eu vou te falar assim: tem o lado da criança, que é difícil para ela porque ela está assustada, porque ela está com dor, está triste, e tem o nosso lado profissional, porque a gente também tem aquela tensão. Então, eu acho que o uso do lúdico e do brincar alivia os dois lados, as duas partes. Eu acho que ameniza aquele momento para mim e também para a criança.* (Cantarolar)

Subtema: Contexto organizacional

Na percepção das participantes, a instituição não incentivava a utilização do brincar e do lúdico pelas técnicas de enfermagem, além de não valorizar a ambiência lúdica da unidade. Significaram isso como desdobramentos da abordagem centrada na doença e da preocupação com a contenção de gastos presentes na instituição. Isso fez com que as participantes mobilizassem, por si mesmas, estratégias para que o lúdico e o brincar se fizessem presentes em suas práticas e no cotidiano de trabalho.

*Por parte da instituição, a gente não tem incentivo, tanto que quem corre atrás das coisas* (lúdicas) *é a gente, quem decora aqui é a gente comprando tudo do nosso dinheiro. A gente tira dinheiro do nosso bolso para poder deixar o ambiente um pouco melhor.* (Boneca)

*Eu entendo que o técnico de enfermagem pode usar o lúdico e o brincar, mas aqui, nessa instituição, nós não temos esse estímulo*. [...] *acredito que seja um problema mais da cultura institucional que foca muito no processo de doença, não foca tanto no processo do ser humano da criança, da família.* (Queimada)

*Não tem incentivo nenhum por parte da instituição e nem oferta de brincadeiras ou de brinquedos.* [...] *isso atuou demais como desestimulador* (para o uso do brincar/lúdico). (Casinha)

Para além do exposto, a maioria das entrevistadas relatou não ter sido o brincar, o lúdico e a brinquedoteca temas abordados pela instituição em termos de educação continuada ou orientação para suas práticas. Comentou ainda que a relação com as tecnologias e o ambiente acontecia de forma intuitiva.

*De quando eu entrei na pediatria, nunca recebi nenhum treinamento ou orientação relacionado ao brincar, ao lúdico ou ao uso da brinquedoteca. Eu acho que seria muito importante.* (Amiguinha)

*Olha, eu estou aqui já faz um ano e oito meses, e eu não tive nenhum treinamento até hoje. Aqui, os cursos são mais voltados às técnicas e procedimentos, mas essa parte do lúdico, do brincar, da humanização, isso aí eu não vi.* (Pular corda)

*Nós temos vários treinamentos sobre a técnica, mas não dessa questão que poderia facilitar a técnica. E eu acho que como muitas pessoas não colocam isso em prática, seria uma coisa que valeria a pena fazer esse tipo de incentivo, porque não é incentivado, não é elencado isso como uma coisa de importância.* (Elefantinho colorido)

Destarte, o espaço físico da unidade pediátrica, com existência de brinquedoteca/brinquedos, foi exaltado pelas participantes do estudo, contribuindo com a adoção deles em suas práticas, caso desejassem realizá-la.

*Eu acho que o espaço ali é muito bom, é grande. Às vezes, a gente interage com as crianças jogando bola ali, no meio daquele saguão, às vezes na brinquedoteca.* (Pula-pula)

*Dá para usar o brincar e o brinquedo nos quartos, no pátio, que tem aquela área de lazer enorme, e na brinquedoteca também.* (Amarelinha)

Diante desse cenário, a brinquedoteca foi reconhecida pelas participantes como sendo promotora da incorporação dos recursos que lá estão em suas ações profissionais, atuando na recuperação da criança e proporcionando que a experiência da internação não seja tão traumática, mesmo que seu uso não fosse estimulado pela instituição.

*É um lugar* (brinquedoteca) *que transmite leveza no trabalho da gente aqui. Às vezes, eu vou ali, mexo em alguma coisa, levo uma criança. Eu vejo que ela transmite leveza num ambiente pesado, que é o ambiente hospitalar. Ali é o único espaço onde ela lembra que ela é uma criança. Então, eu acho que a brinquedoteca tem um papel muito importante para dar um ânimo para a criança.* (Boneca)

*Os técnicos não são estimulados a utilizar a brinquedoteca; a iniciativa é individual de cada profissional. Alguns têm mais iniciativa e outros têm menos*. [...] *eu acredito que deveria ao menos haver um treinamento da equipe de enfermagem que vai trabalhar na pediatria ou em qualquer lugar onde houver a presença das crianças.* (Queimada)

## DISCUSSÃO

Os significados e as ações de uso do lúdico e do brincar emergem e se amoldam a partir do processo interacional desenvolvido entre técnicas de enfermagem e crianças hospitalizadas, sendo envolto e influenciado pelo contexto organizacional. As perspectivas utilizadas para definir a prática lúdica e o brincar enquanto elementos essenciais no cuidado à criança hospitalizada derivam de experiências e crenças profissionais e pessoais, internalizadas pelas técnicas, que as relacionam à humanização e redução de possíveis traumas.

A hospitalização é considerada uma experiência complexa para a criança, com potencial estressor e traumático, assim como é para seu acompanhante. Ambos são expostos a um ambiente desconhecido e têm sua rotina modificada^(2,19)^. Tal fato foi relatado por grande parte das participantes desta pesquisa, as quais significaram que o brincar surge como mediador de experiências mais positivas durante a estadia da criança no hospital. Isso demonstra a necessidade de que as instituições invistam na ambiência da unidade pediátrica, com o intuito de tornar aquele ambiente mais acolhedor e confortável para seus pacientes^(1)^ e, consequentemente, promova e sustente a cultura do brincar na unidade.

Atualmente, sabe-se que o brincar e o lúdico conferem vários benefícios durante a hospitalização da criança, entre eles: minimiza os sentimentos de tristeza ou traz alegria; auxilia na sua recuperação; facilita a interação com o profissional; minimiza a dor e o desconforto durante o procedimento; provoca estímulos sensoriais; deixa a criança mais tranquila e menos estressada; facilita a aceitação do procedimento; propicia a criação de vínculo; e fortalece a confiança no profissional^(11,20)^. Todos os desdobramentos apontados na literatura também foram discutidos pelas participantes deste estudo.

Vale a pena destacar que o brincar é um direito da criança e inerente a ela^(21)^. É ainda a linguagem por meio da qual expressa suas necessidades. Sendo assim, a promoção de um cuidado que valoriza o lúdico oportuniza a alegria do “ser criança”. Isso corrobora a visão das participantes do presente estudo de que a criança, enquanto pessoa, necessita de um olhar diferente do adulto, com capacidade para revelar percepções e necessidades. Para a equipe de enfermagem, quando o profissional se dispõe a brincar, além de atender à necessidade da criança e favorecer a interação, ele equipara-se a seu nível de pensamento, construindo e reconstruindo seus significados e promovendo mudanças comportamentais^(13)^, visando um cuidado humanizado^(4)^. Elas entendem que esse movimento igualitário torna a situação interessante à criança, que prontamente transforma essa oportunidade em possibilidade para o brincar compartilhado com o profissional^(4,13)^. Ainda, faz com que as crianças possam expressar seus sentimentos e emoções, além de compreender melhor o processo e a necessidade de hospitalização^(5,13)^_._

De acordo com esse e demais estudos^(4,5,9)^, o uso do lúdico e do brincar facilita também a realização de procedimentos de enfermagem, especialmente em técnicas como punção e/ou administração de medicamentos intravenosos, uma vez que as crianças ficam mais calmas e relaxadas, conseguindo cooperar mais com o profissional. Além disso, tais práticas promovem o estreitamento de vínculos, favorecem a recuperação e ajudam no alívio da ansiedade e tensão provocadas pela internação^(5,9,22)^.

Por outro lado, parte das participantes entrevistadas significou que o uso do lúdico e do brincar faz parte das práticas assistenciais de outros profissionais, sendo sua função a realização de procedimentos e execução da prescrição médica. Tal atitude vai de encontro aos resultados encontrados na literatura, os quais apontam para a concepção de ser o brincar um fazer “extra” não integrante da responsabilidade da enfermagem, e que não conta no rol de suas competências^(7,9,23)^. Logo, percebe-se que o brincar não está incluído na enfermagem, tendo um papel secundário e informal, voluntário e não institucionalizado, que não faz parte do processo de cuidado de enfermagem à criança. Assim, reforça-se a necessidade de que as técnicas de enfermagem ressignifiquem o uso desses recursos, provocando mudanças atitudinais^(13)^, que incentivem a assunção do brincar como própria da enfermagem. Chama a atenção a vinculação da profissional ao médico, apesar de pertencer à equipe de enfermagem, aspecto interessante de ser explorado em futuros estudos. Ainda, fica a reflexão acerca das prescrições de enfermagem não se direcionarem ao brincar.

De todo modo, houve participantes que demonstraram atitudes positivas em relação ao brincar e ao lúdico. Assim como demonstrado em outros estudos^(4,7,9,22)^, as técnicas de enfermagem acreditam que esses recursos conferem benefícios durante a hospitalização da criança e, por isso, desenvolveram a percepção de que são essenciais para a recuperação, o bem-estar e a felicidade de seus pacientes^(24)^. Sendo assim, apoiam e defendem seu uso^(11)^, buscando sustentá-los, a exemplo daquelas que trazem recursos para poder utilizar no seu fazer profissional, uma vez não contarem com pleno suporte institucional para sua incorporação. Questiona-se, então: como instituições com unidades pediátricas estão a considerar o brincar e a brincadeira na agenda de seus custos?

Ao discutir sobre os favorecedores do uso do lúdico e do brincar nas práticas das técnicas de enfermagem, notou-se a existência de fatores intrínsecos e extrínsecos aos profissionais^(9)^. Como fatores intrínsecos, citam-se a promoção da recuperação da criança e o auxílio para lidar com o estresse da hospitalização, o que as faz sentir motivadas e satisfeitas. De acordo com as entrevistadas, ser alegre, divertida e brincar não trazia benefícios somente para a criança, mas também para elas mesmas, o que estimulava ainda mais o uso dessas práticas, resultado similar ao encontrado na literatura^(7,9,22,23)^. Reconhecem ainda que a criança, ao brincar com o enfermeiro, (re)significa a imagem dele para alguém mais acolhedor e brincante^(7,13)^.

Já os fatores extrínsecos estão associados à presença da brinquedoteca e demais recursos lá existentes^(9)^. A brinquedoteca hospitalar auxilia a criança a passar pelo processo de internação adquirindo confiança, facilitando a adaptação e minimizando seu sofrimento. É um espaço no qual ela se aproxima da rotina que tinha fora do hospital. Para a equipe de enfermagem, pode auxiliar na aproximação com a criança e seus familiares, facilitando os procedimentos e exames, tornando-os menos dolorosos e contribuindo para a adesão ao tratamento, podendo aumentar as possibilidades de recuperação^(25)^. No presente estudo, ficou evidente que a existência de um amplo espaço na pediatria simbolizou um motivador ao uso do lúdico e do brincar. Entretanto, a falta de incentivo da instituição para exploração do ambiente acarretou um fator desanimador para envolvimento nas atividades lúdicas. Logo, fica evidente a necessidade de que as instituições ressignifiquem a pertença e inserção do brincar, do lúdico e da brinquedoteca no fazer profissional da equipe de enfermagem, com efetivo apoio e reconhecimento desse tipo de intervenção^(13)^.

Outro fator que atuou como desmotivador no presente estudo foi a ausência de indução e orientação institucional, principalmente quanto à oferta de formações profissionais que abordassem o lúdico e o brincar com a criança, o que as tornaria mais seguras e confiantes para atuar na pediatria. Soma-se a isso as lacunas enfrentadas durante o curso técnico em enfermagem, o qual as orientava a não estabelecerem vínculos com seus pacientes. Esse aspecto requer renovação, com indicação de ser objeto de estudos futuros.

Outras pesquisas realizadas revelaram que a demanda de trabalho, a rotina dos procedimentos, o fazer mecânico dos cuidados, a falta de tempo, a escassez de infraestrutura e formação continuada estiveram apontadas como determinantes para a pouca viabilidade de seu uso pela equipe de enfermagem, com questionamentos acerca do lugar do brincar na cultura institucional^(7,11,26)^. Ademais, outro estudo realizado^(4)^ também reforçou esses achados, revelando a não incorporação do brincar ao processo de enfermagem nas instituições, reforçando o não reconhecimento da díade brincar-cuidar, um avanço que precisa tornar-se agenda da atenção à saúde, sobretudo no cenário pediátrico.

Torna-se importante pontuar também a importância do papel do enfermeiro no atitudinal de sua equipe. Ele deve ser capacitado para desenvolver o brincar e o lúdico de forma intencionada, planejada e integrada ao plano de cuidados, além de incentivar e manter sua equipe capacitada para o cuidado, levando-as a uma mudança nos significados atribuídos, até então, a essas tecnologias, que ao serem vivenciados nas interações sociais, provocam mudanças de comportamento^(7,13)^. Em nosso estudo, foi demonstrado que a presença de um enfermeiro o qual estimula sua equipe faz com que elas se sintam mais dispostas e motivadas a inserir esses recursos em suas práticas.

Logo, fica evidente que as autoridades e os gestores do hospital precisam estar cientes da importância do brincar e do lúdico para crianças internadas, com o intuito de prestar todo seu apoio à implementação de atividades lúdicas e fornecer os recursos necessários para facilitar a integração e utilização da brincadeira como uma estratégia para promover o conforto, aliviar a ansiedade, permitir a comunicação e a expressão de medos, além de promover a recuperação e o bem-estar geral da criança hospitalizada^(9)^. Esse cuidado institucional cria uma cultura e estimula, nas interações sociais^(13)^, o compartilhamento da singularidade dos recursos para a humanização e acolhimento de necessidades, a exemplo do brincar para a criança.

Esta pesquisa, ao dar voz às técnicas de enfermagem atuantes em unidade pediátrica, busca sensibilizar profissionais e gestores dessas instituições acerca da importância e dos benefícios da utilização do lúdico e do brincar, com possibilidade de provocar mudanças e ressignificações^(13)^ que promovam a humanização do cuidado pediátrico, com apostas junto a essa categoria profissional. Como limitantes deste estudo, encontra-se o fato de ser desenvolvido em uma única realidade hospitalar, com parte significativa das participantes deixando de integrar a segunda etapa da pesquisa. Ademais, o fato de as entrevistas acontecerem de forma presencial e *online* também pode atuar como limitador, uma vez que ocorrem diferentes mediações, explorações e presenças. Entretanto, por ser uma escolha da participante, acredita-se que optaram pela forma com a qual se sentiam mais confortável, o que favoreceu as narrativas.

O referencial do IS foi potente para focalizar os comportamentos das participantes assinalando os significados que os estruturavam, assim como os elementos da cena social que mobilizavam processos no *self*. As interações com a criança sobressaíram nesse contexto, merecendo destaque também o processo de desenvolvimento de uma consciência sobre “si mesmo” enquanto técnica de enfermagem, a partir de um processo de reflexibilidade que se “indaga” acerca do que está “socialmente” e institucionalmente posto à profissão. Esse processo explicita a emergência do “mim” atuando na consciência do si, com contribuições à tomada de decisão acerca da incorporação do brincar e do lúdico.

## CONCLUSÃO

Este estudo possibilitou conhecer as percepções de técnicas de enfermagem atuantes em uma unidade de internação pediátrica acerca do uso do lúdico e do brincar em suas práticas profissionais. Percebeu-se a prevalência do simbolismo de serem os recursos contribuintes para a execução de procedimentos técnicos e formação de vínculo. Porém, os resultados apontaram para um contexto pouco sustentador desse uso e indutor do significado de serem as tecnologias do escopo de outros profissionais. Houve participantes que reconheceram e significaram os recursos como alinhados a um cuidado humanizado, com esforços de investirem e manterem comportamentos que os incorporem.

Dessa forma, conclui-se que há necessidade de investir na discussão sobre a importância de apostar nos técnicos de enfermagem enquanto categoria que pode contribuir e promover o lúdico e o brincar durante a hospitalização. Para tanto, renovações nos posicionamentos institucionais se fazem urgentes, com destaque para aquelas envolvidas com a formação desses profissionais, suas empregadoras e o conselho de classe. Técnicos de enfermagem podem ampliar e sustentar a presença do lúdico e do brincar no cotidiano da hospitalização infantil. O brincar e o brinquedo são instrumentos terapêuticos nesse cenário; o fornecimento de infraestrutura e suporte para amplo uso deles é fundamental; e a formação profissional e continuada precisa reconhecer e abordar o tema dentro do que é pertinente a cada profissão. De todo modo, um bom começo é que os centros de formação técnica em enfermagem abordem essa temática, desenvolvendo habilidades e competências para um uso dentro do seu escopo profissional, com contribuições à qualidade do cuidado ofertado em unidades pediátricas hospitalares.

## Data Availability

O conjunto de dados que dá suporte aos resultados deste estudo não está disponível publicamente.

# Translation: Percepciones de las técnicas de enfermería sobre el uso del juego y el entretenimiento en las prácticas profesionales

**DOI:** 10.1590/1980-220X-REEUSP-2025-0024es

**Authors:** Gabriele Petruccelli, Monika Wernet, Aline Oliveira Silveira, Edmara Bazoni Soares Maia, Cristiane Leite de Almeida, Fernanda Manuela Loureiro

**Affiliations:** 1 Universidade Federal de São Carlos, Programa de Pós-Graduação em Enfermagem, São Carlos, SP, Brasil.; 2 Universidade Federal de São Carlos, Departamento de Enfermagem, São Carlos, SP, Brasil.; 3 Universidade de Brasília, Departamento de Enfermagem, Brasília, DF, Brasil.; 4 Universidade Federal de São Paulo, Escola Paulista de Enfermagem, São Paulo, SP, Brasil.; 5 Escola Técnica Estadual Paulino Botelho, São Carlos, SP, Brasil.; 6 Universidade Católica Portuguesa, Lisboa, Portugal.

**Keywords:** Enfermería Pediátrica, Hospitalización, Niño, Juego e Implementos de Juego, Enfermeros no Diplomados

## Abstract

**Objetivo:**

Comprender las percepciones de las técnicas de enfermería de una unidad de internación pediátrica sobre el uso del juego en sus prácticas profesionales.

**Método:**

Estudio exploratorio con abordaje cualitativo, sustentado en el referencial teórico del Interaccionismo Simbólico, desarrollado en una unidad pediátrica de un hospital universitario del interior de São Paulo. La recolección de datos se realizó de marzo a diciembre de 2024, mediante entrevistas semiestructuradas, con 14 participantes. El análisis temático reflexivo apoyó la evaluación de los datos.

**Resultados:**

Los temas “Propósitos de la adopción de actividades lúdicas y recreativas” y “Determinantes de la adopción de actividades lúdicas y recreativas” revelaron que las técnicas de enfermería vinculan los recursos a la ejecución de procedimientos técnicos y a la formación de vínculos. Sin embargo, pusieron de relieve un contexto poco favorable a este uso y que llevó a que el significado de estos recursos quedara al alcance de otros profesionales.

**Conclusión:**

El uso del juego y actividades recreativas predominó en la ejecución de los procedimientos, teniendo un lugar secundario, informal, voluntario y no institucionalizado en la atención de enfermería al niño.

## INTRODUCCIÓN

La hospitalización es un acontecimiento complejo para los niños, generando estrés, ansiedad, sufrimiento, angustia y tensión, con el potencial de convertirse en una experiencia traumática. Además, tanto el niño como su acompañante están expuestos a lo desconocido, ya que su vida cotidiana se ve alterada^(1,2)^.

Además, durante el periodo de hospitalización, el niño puede estar expuesto a procedimientos diagnósticos y terapéuticos potencialmente dolorosos, así como aislado de su familia, amigos y entorno educativo^(1,3)^.

En este escenario, es frecuente que se enfrenten a restricciones en el juego, una ocupación esencial^(1,3)^. Se ha demostrado que la incorporación de juegos y actividades lúdicas transforma el entorno hospitalario, sobre todo al simbolizar lo que es familiar para el niño^(3,4)^, aportando una sensación de normalidad y continuidad a su vida. Esto significa que estos recursos actúan como válvula de escape, ayudando a desarrollar estrategias de afrontamiento de la enfermedad y de la hospitalización, favoreciendo la distracción y el entretenimiento, facilitando la asimilación de la información y reduciendo los niveles de ansiedad^(3,4,5,6,7)^.

Es necesario resaltar que, además de ser un derecho del niño^(8)^, el uso del juego tiene un carácter terapéutico y deja de ser un mero pasatiempo, siendo capaz de actuar sobre el estado clínico del niño y promover su recuperación. También es capaz de reforzar los vínculos entre el profesional, el niño y su cuidador^(4,5,6)^. La incorporación de estas tecnologías en salud favorece una atención integral y humanizada, centrada en el niño y que apoya su autonomía^(3,5,6)^. Por ello, es urgente que estos recursos sean utilizados de forma intencionada y recurrente por los profesionales que intervienen en su cuidado en el contexto de la hospitalización infantil.

Actualmente, se sabe que el personal de enfermería incorpora el uso del juego y las actividades lúdicas a través de lenguajes lúdicos, muñecos, juegos y juguetes como mediadores de las interacciones, además de hacer uso de uniformes coloridos y parafernalia infantil^(4)^. Es importante destacar que, a pesar de las evidencias científicas que comprueban la importancia del uso del juego y las actividades lúdicas^(4,6,9,10)^ en las prácticas de los profesionales de enfermería, su adopción es aún lacunar e incipiente^(4,7,10,11)^, siendo escasas las evidencias específicas para los técnicos de enfermería.

Como consecuencia de esta fragilidad en el uso del juego, el establecimiento de interacciones con el niño es limitado, así como la construcción de vínculos terapéuticos. Esto lleva al niño a crear una imagen negativa del técnico de enfermería, reduciéndolo a la realización de procedimientos dolorosos y desagradables^(4,7)^. Por estas razones, es fundamental que el juego se incorpore al proceso de trabajo de enfermería y presida la relación entre el profesional y el niño hospitalizado, asegurando su potencial terapéutico^(4,7)^, promoviendo experiencias de hospitalización menos traumáticas.

En este sentido, existe un vacío en la literatura a la hora de dar voz a los técnicos de enfermería, que, en Brasil, representan una categoría profesional que está constantemente en contacto directo con los niños hospitalizados. Por lo tanto, este artículo se propone analizar las prácticas de los técnicos de enfermería que trabajan en una unidad hospitalaria pediátrica. La cuestión es: ¿cómo los técnicos de enfermería de las unidades hospitalarias pediátricas perciben y adoptan el juego en los cuidados de enfermería? El objetivo fue conocer las percepciones de los técnicos de enfermería de una unidad de hospitalización pediátrica sobre el uso del juego en sus prácticas profesionales.

## MÉTODO

### Tipo de Estudio

Se trata de una investigación exploratoria con enfoque cualitativo, basada en el marco teórico del Interaccionismo Simbólico (IS), centrado en los comportamientos, significados, sentidos, valores y creencias^(12)^ relacionados con el juego y las actividades lúdicas en las prácticas de los técnicos de enfermería con niños hospitalizados. El IS entiende que los significados resultan de las interacciones sociales. Los individuos perciben los hechos que les rodean y evalúan y transforman significados sobre ellos, culminando en la emergencia de acciones y comportamientos^(13)^. El proceso interaccional es continuo y, por tanto, la comprensión y el significado de las cosas son abiertos. En este manuscrito se adoptó la pauta propuesta por los *COnsolidated criteria for REporting Qualitative research* ^(14)^.

### Sitio del Estudio

Este estudio se realizó en un municipio del centro-este del interior del estado de São Paulo, con una población estimada de 254.857 habitantes, de los cuales 16,97% tenían entre 0 y 14 años^(15)^. El escenario de la investigación fue la unidad de internación pediátrica del hospital universitario de la ciudad, que es una de las referencias de la región en internación pediátrica. La unidad cuenta con 12 camas de hospitalización para niños de hasta 12 años^(16)^.

### Población y Criterios de Selección

En el momento de esta investigación, el equipo de enfermería estaba formado por 27 técnicos de enfermería y 13 enfermeras, estas últimas divididas entre cuidados y gestión. La jornada laboral de todo el equipo de enfermería era de 36 horas semanales, divididas en turnos de 12 horas. Los criterios de inclusión fueron ser técnico de enfermería que trabajara en la unidad de pediatría y haber trabajado en ella durante al menos un mes. Los criterios de exclusión fueron haber estado fuera de la unidad durante más de tres meses durante el periodo en el que la investigadora estuvo sobre el terreno.

Se invitó a todos los técnicos de enfermería a participar en la investigación. Dos no aceptaron porque, según ellos, había muchas investigaciones en curso en el hospital y no veían la necesidad de participar en ninguna más. El primer autor recogió personalmente los datos durante las horas de trabajo. La entrevista se programó por correo electrónico. Un total de 14 técnicos devolvieron los mensajes y aceptaron participar en las entrevistas; dos habían abandonado la institución; ocho no respondieron a los intentos de ponerse en contacto con ellos; y uno contestó que no estaba disponible. El número de participantes se basó en una muestra dada previamente, pero los datos fueron suficientes, ya que se llegó a los conceptos y sus relaciones para comprender el fenómeno explorado.

### Recolección de Datos

Para la recolección de los datos se utilizaron dos estrategias: la observación no participante y las entrevistas semiestructuradas, todas realizadas por la primera autora. En este artículo se discuten los resultados obtenidos a partir de las entrevistas con técnicos de enfermería. Esta estrategia se desarrolló a partir de un conjunto de preguntas abiertas previamente formuladas. Cabe destacar que, a pesar de la existencia de un guión, este tipo de entrevista necesita estar abierta a la exploración de los pensamientos, sentimientos y creencias de los participantes sobre los fenómenos tratados^(17)^.

Las entrevistas se realizaron entre marzo y diciembre de 2024 y tuvieron lugar en días y horas acordados con los participantes. Tuvieron lugar tanto en persona como virtualmente, mediadas por la plataforma *Google Meet* ^®^, según la disponibilidad de los entrevistados. Las preguntas a utilizar fueron discutidas y revisadas en el grupo de investigación al que pertenecen las autoras, además de ser sometidas al análisis de enfermeras pediátricas que trabajan con la temática abordada en este estudio.

Como disparador inicial, se formuló la siguiente pregunta: ¿cómo percibe usted el uso del juego y de lo lúdico en sus prácticas con los niños? A esta pregunta le siguieron otras: ¿cómo cree que surgió esta forma de pensar/actuar? ¿Cuál es el significado del juego para ti como técnico de enfermería? ¿Cómo se relacionan el juego y lo lúdico con tu práctica profesional? Pensando en la unidad de pediatría del hospital universitario, dime qué te motiva a utilizar el juego y las actividades lúdicas. En la misma línea, dime qué le disuade de utilizar el juego. Si el participante discutía la intención de una de las preguntas, no se presentaba. Del mismo modo, si hacía afirmaciones que podían explorarse con otras preguntas, se presentaba.

La duración media de las entrevistas fue de aproximadamente 32 minutos. Tanto las entrevistas presenciales como las virtuales se grabaron en audio con un dispositivo electrónico y se transcribieron íntegramente con *el programa Transkriptor* ^®^. Las transcripciones se revisaron dos veces para validar el material. Las transcripciones no fueron validadas por los participantes.

### Análisis y Tratamiento de los Datos

Los datos obtenidos se analizaron al mismo tiempo que las entrevistas y se basaron en el análisis temático reflexivo propuesto por Braun y Clarke^(18)^. Este proceso analítico implicó la familiarización con los datos, en la que se leyeron repetidamente las transcripciones, destacando los elementos asociados al fenómeno analizado. A continuación se procedió a la codificación, en la que las transcripciones se codificaron a la luz de la pregunta y el objetivo del estudio. En la fase de búsqueda de temas, los códigos obtenidos en la fase anterior se agruparon en temas. En la fase de revisión de temas, éstos se validaron. A continuación, se definieron y nombraron los temas y se redactó un análisis detallado de cada uno de ellos. Por último, se redactó el borrador final, que integraba la narrativa analítica en su conjunto junto con la bibliografía^(18)^. Todo el análisis fue realizado por el primer autor, luego con el segundo autor y, en una tercera etapa, discutido con los demás.

### Aspectos Éticos

El estudio fue sometido y aprobado por el Comité de Ética de la Investigación en Seres Humanos, con el Dictamen nº 5.983.460 y el Certificado de Sometimiento a Evaluación Ética nº 67333623.0.0000.5504. Se respetaron todos los preceptos éticos de acuerdo con la Resolución 510 de 2016. Cabe resaltar que se cumplió con todo el rigor científico, incluyendo la lectura y firma conjunta del Formato de Consentimiento Informado. Se preservó la identidad de los participantes, quienes fueron identificados con el nombre del juguete/juguete que más les gustaba en la infancia, a su elección.

## RESULTADOS

Se entrevistó a catorce técnicos de enfermería, todas mujeres, pertenecientes a cuatro turnos, dos de ellos diurnos y dos nocturnos. La edad mínima era de 33 años y la máxima de 58, con una media aproximada de 46 años. La duración media de la formación como técnico de enfermería era de 18 años, con un mínimo de diez y un máximo de 28 años. El tiempo de trabajo en pediatría osciló entre un año y cuatro meses y 21 años, mientras que el tiempo de trabajo en el servicio de pediatría incluido en el estudio osciló entre un año y cuatro meses y nueve años.

Los comportamientos de los técnicos de enfermería en relación a las actividades lúdicas y al juego se sustentaron en dos significados: (1) su adopción está en consonancia con las prácticas humanizadas; y (2) su adopción favorece la ejecución de los procedimientos de enfermería. Estos significados fueron desplegados por los atributos personales de los profesionales, los resultados experimentados a través del uso de recursos, el contexto organizacional y las interacciones durante la formación profesional, así como las experiencias con niños de su entorno.

Los temas y subtemas obtenidos del análisis de los datos (Cuadro 1) cubren el ámbito de este estudio en cuanto a las percepciones de los técnicos de enfermería de una unidad hospitalaria pediátrica sobre el uso del juego en sus prácticas profesionales.

**Cuadro 1:** Codificación de los temas y subtemas derivados del análisis de los datos obtenidos en el estudio – São Carlos, SP, Brasil, 2024.

Tema	Subtemas
Finalidades de la adopción del juego y lo lúdico	Realización de procedimientos Humanización de las practices
Factores determinantes de la adopción del juego y la ludificación	Formación professional Interacciones com los niños Atributos personales Resultados del uso Contexto organizativo

Fuente: propia autoria, 2024.

Tema: Finalidades de la adopción del juego

Subtema: Realización de procedimientos

Los técnicos de enfermería apoyaron la conducta de utilizar el juego por las contribuciones que aporta a la realización de procedimientos técnicos, entendidos como finalidad de su profesión. Para ellos, las ventajas se relacionaron principalmente con la reducción del tiempo necesario para realizarlos, debido a la colaboración lograda con el niño.

*La mayor motivación para mí es agilizar mi proceso, el propósito que tengo cuando entro en la habitación del niño. La mayor ventaja es el tema de la optimización de la demanda de tiempo, porque agiliza mi atención, gano más tiempo.* (Rayuela)

*A veces las personas hacen de su profesión algo mecanicista, como si fuera una línea de producción, y piensan que haciendo esto les va a quitar tiempo, cuando en realidad les va a ahorrar tiempo, porque el procedimiento se va a volver más fácil y rápido de hacer.* (Elefantito de colores)

También fue relatado que el uso del juego favoreció la ejecución de los procedimientos, fortaleciendo la relación con el niño, promoviendo distracción y relajación durante el procedimiento, además de permitir la transmisión de informaciones sobre la técnica. Esto garantizaba la seguridad y comodidad del niño, aliviando los temores del paciente, incluso los del profesional.

*El juego ayuda a los niños a establecer vínculos con el equipo de enfermería. Cuando el niño refuerza el vínculo con el profesional a través del juego, nos resulta mucho más fácil llevar a cabo la rutina de cuidados. El juego ayuda al niño a relajarse mucho, le ayuda a entender su proceso, a sufrir menos con los procedimientos.* (Quemada)

*Creo que cuando juegas con un niño, te estás igualando a ellos. Así que te miran y piensan: “Vaya, es una niña como yo, eso es genial, puedo confiar en ella”.* (Saltando a la cuerda)

*Me doy cuenta de que cuando jugamos a algún tipo de juego con el niño, nos inventamos una historia, hablamos, decimos algo ajeno a lo que están viviendo, se desvía la atención de ese momento y empiezan a imaginar otra cosa. Entonces, es más fácil y menos doloroso para ellos pasar por ese procedimiento, y es más fácil para nosotros también, porque podemos, sobre todo cuando se trata de punciones, equivocarnos menos, porque el niño va a mover menos el brazo, lo vas a pinchar menos veces.* (Peteca)

Al ejemplificar los aportes relacionados con la realización de los procedimientos, destacaron que no hay éxito cuando se realizan de forma mecanizada, ya que asustan y desestabilizan al niño, aspectos que repercuten en el tiempo de realización de los procedimientos.

*Hay que tener cuidado: jugar, hablar, tener paciencia. Porque a veces estamos apurados, queremos hacer los procedimientos rápido y no podemos.* (Rompecabezas)

*Me di cuenta de que cuando no usamos nada y sólo hacemos los procedimientos de forma automática, el niño empieza a llorar, y entonces tardamos más en hacer las cosas.* (Rayuela)

A pesar de eso, hubo participantes que reconocieron que no utilizaban el juego y las actividades lúdicas en sus prácticas, porque sentían que estaban encargados de realizar los procedimientos y no de utilizar esos recursos. En este contexto, había quienes consideraban que estas actividades debían ser dirigidas a otro profesional, y que su cometido era cumplir con las prescripciones médicas. Además, entendían que incorporar estas actitudes lúdicas retrasaría, en general, la realización de sus tareas.

*No suelo utilizarlo porque realmente no tenemos tiempo para ello. Cuando estoy de guardia, soy responsable de más de un niño, y hay varios procedimientos. Y no creo que sea ideal utilizar el juego en este momento para el técnico de enfermería. Como he dicho, tengo mis obligaciones y tengo que cumplirlas, tengo que ocuparme de ellos, porque tenemos una prescripción médica que tenemos que cumplir. Y eso nos quita horas de trabajo. Así que no me voy a preguntar qué es lo que no voy a poder hacer. Eso lo tiene que hacer otro profesional, no el mismo profesional que está haciendo la atención.* (Tarareando)

*Como todavía soy nueva en pediatría, no tengo la habilidad que tiene la gente para hacer las cosas muy rápido. Así que a veces pienso que si paro un minuto para jugar, voy a llegar tarde, y entre jugar y llegar tarde, prefiero no jugar y hacer las cosas bien.* (Dibujo)

Subtema: Prácticas humanizadoras

Para los técnicos de enfermería, el juego, los juguetes y lo lúdico estaban vinculados a ‘ser niño’, incluso en el contexto de la hospitalización, y eran importantes en la relación con el paciente desde el momento del ingreso hasta el alta.

*Para mí, como técnico, el juguete es algo que salva, porque es muy importante para el niño. Creo que un niño sin juguete no es un niño, y eso es lo que tiene un niño, reír, jugar.* (Rompecabezas)

*Ya me he dado cuenta de que el juego, lo lúdico, forma parte de todo, desde el ingreso del niño al tratamiento, hasta que el niño se va, celebrando, jugando, celebrando con ellos cuando se van. Creo que esto tiene que ocurrir en pediatría, porque es como entrar en su pequeño mundo. (Stop)*

Para los participantes, reconocer al niño como una persona con derechos significaba que se esforzaban por interactuar con ellos, garantizándoles voz y un lugar bajo el confort relacional. En este sentido, su comportamiento consistía en escuchar y hablar intencionadamente con el paciente, bajo la mediación del juego. Las conversaciones pretendían que el niño comprendiera lo que iba a ocurrir, especialmente cuando se llevaban a cabo procedimientos.

*La gente dice: “Ah, es que es un niño”, pero a mí me gusta mucho explicarles todo el procedimiento que voy a hacer de forma que lo entiendan.* (Dibujo)

*A veces, dependiendo del tamaño del niño, me bajo a hablar con ellos, para que se sientan más tranquilos al hablar. (Stop)*

*Es una forma de comprensión, de cariño también, porque si digo: “Voy a clavar una aguja”, el niño se va a asustar. Tampoco podemos decir que no va a doler. Decimos que va a picar y todo eso, que es una hormiguita, algo para que no se asusten tanto.* (Barbie)

Algunos de los técnicos de enfermería reconocieron la hospitalización como un acontecimiento traumático y hostil para el niño, al encontrarse fuera de su entorno familiar y bajo un control restringido de la situación. En este escenario, veían el juego como facilitador y mediador de experiencias más positivas.

*La niña ha sido colocada en un entorno hostil, con desconocidos que la manipulan. Ya está en un entorno fuera de su casa; ya está enferma; ya está en un momento delicado.* (Muñeca)

*Así que intentas, en la experiencia, quitar ese momento, quitar esa tensión, porque todo el mundo está fuera de tu entorno, de tu casa, de tu pequeña habitación. Entonces tratamos de aliviarlo lo más posible para que el niño no tenga tantos traumas.* (Peteca)

A la luz de esto, según los técnicos de enfermería, el juego y las actividades lúdicas en sus prácticas estaban relacionados con la promoción de la sonrisa y el bienestar del niño, favoreciendo su recuperación.

*La mayor ventaja de utilizar el juego, creo, está más asociada al paciente, al sentimiento que despierta en el niño. El niño es más feliz, podemos ver que son felices.* (Rayuela)

*Lo que me motiva a utilizar el juego es el bienestar de los niños. Ver una sonrisa en sus caras. Para mí, eso significa amor.* (Saltitos)

*Veo* (el juego) *como algo positivo para el niño. Les ayuda a mejorar mucho. Así que si se sienten más acogidos, más queridos, más importantes, mejorarán más rápido.* (Casita)

Tema: Determinantes de la adopción de juegos y actividades lúdicas

Subtema: Formación profesional

Según los relatos de los participantes, durante el curso técnico les dijeron que el papel del técnico de enfermería era realizar procedimientos y que no debían involucrarse con la persona a la que cuidaban. Sin embargo, cuando experimentaron interacciones en la unidad pediátrica, los significados que les habían enseñado se relativizaron y empezaron a ver la implicación con el niño y el uso del juego como posibilidades para su trabajo.

*Mi formación académica no abordó este tema conmigo* (juego y lúdica)*. Simplemente entras en una planta de pediatría y te das cuenta de que todo es más colorido, todo está más dibujado. Así que intuitivamente deducimos que debería haber más juego y más uso del juego y de los juguetes.* (Quemada)

*Durante mi formación me decían: “No te metas”, pero es imposible que no te metas. Creo que para dar un buen trato allí* (en la unidad pediátrica), *para dar unos buenos cuidados, creo que hay que implicarse.* (Dibujo)

*Cuando hice el curso* (de técnico de enfermería*), los profesores decían: “No puedes crear un vínculo con el paciente, no puedes hablar así, no puedes hablar así, tienes que llegar y hacerlo”. Y me di cuenta de que no es así como funciona en la práctica.* (Muñeca)

Subtema: Interacciones con los niños

Las relaciones con los niños, especialmente las vividas en la vida personal y familiar de cada técnico de enfermería, provocaron reflexiones interiorizadas sobre la importancia de tener en cuenta al niño, escucharlo y acogerlo. Como consecuencia, valoran el juego y lo lúdico.

*Mira, mi vida está rodeada de niños. Tengo una hija en casa, así que trato a los niños como la trato a ella aquí en casa. Siempre he estado muy unida a ella, muy unida a mis sobrinas, así que creo que el hecho de que mi casa esté siempre llena de niños me ha facilitado mucho el contacto con los niños aquí en el hospital.* (Dibujo)

*Al principio, estaba muy asustada, porque pensaba mucho en mi hija. Y pensé: “Vaya, debe de ser muy triste tener un hijo en esta situación y todo eso”. Y luego, como madre, me lo tomé con calma, en plan: “Oh, ¿cómo me gustaría que trataran a mi hija?”. Fue más o menos así, y entonces empecé a tratarla así, y simplemente fluyó.* (Barbie)

*Siempre he tenido una buena relación con los niños, siempre me ha resultado muy fácil, incluso antes de empezar a trabajar en enfermería. Me gusta mucho relacionarme con los niños. Así que ya traía esto conmigo y luego me di cuenta de que, a medida que lo aplicaba, mi trabajo se volvía más fácil, y luego seguí mejorando, utilizándolo cada vez más y viendo que el resultado era bueno.* (Elefantito de colores)

Subtema: Atributos personales

En este escenario, ser alegre, divertido y juguetón fueron características personales enumeradas por los participantes, que consideraron que reverberaban en el uso del juego y las actividades lúdicas en las interacciones con los niños. Afirmaron sentirse bien con esta forma de ser cuando trabajan en pediatría, y describieron la recreación de su presencia, lo que les aporta satisfacción y aumenta su deseo de mantener e invertir en este comportamiento.

*Soy una persona alegre desde que me conozco. Creo que este espíritu está dentro de mí desde que nací, ser alegre, hacer feliz a la gente, reír siempre y esas cosas, y me gustan mucho los niños.* (Saltitos)

*Ah, lo uso todo el día* (juego y jocosidad). *Cada vez que tengo contacto, juego con ellos. Canto. Si están llorando, los subo a mi regazo, me burlo de ellos, paseo por el hospital con ellos enseñándoles las plantas del jardín, los juguetes. Hago esto todo el tiempo, todo el día.* (Amiguita)

Subtema: Resultados frente al uso

Al adoptar el juego y las actividades lúdicas en sus prácticas, los técnicos de enfermería relataron haber retroalimentado su comportamiento de uso, una vez que construyeron el significado de demostrar un acto empático y afectuoso que califica su práctica de cuidado, llegando a la persona que estaba con el niño. Así, el uso fue simbolizado como aliviador de la tensión entre el técnico, el niño y el acompañante, promoviendo una relación más próxima y la satisfacción de todos.

*Veo que el estado del niño mejora. Veo la mejoría del niño inmediatamente. Eso es lo que me motiva. Cuanto más reacciona el niño al jugar, más ganas tengo de trabajar con él. El juego y las actividades lúdicas proporcionan esto al niño y al equipo; es bueno para ambos.* (Quemada)

*Te diré esto: está el lado del niño, que es difícil para ellos porque tienen miedo, porque tienen dolor, están tristes, y está nuestro lado profesional, porque también tenemos esa tensión. Así que creo que el uso del juego alivia ambos lados, ambas partes. Creo que alivia ese momento para mí y también para el niño.* (Tarareando)

Subtema: Contexto organizativo

En la percepción de los participantes, la institución no fomentaba el uso del juego por parte de los técnicos de enfermería y no valoraba el ambiente lúdico de la unidad. Consideraban que esto era consecuencia del enfoque centrado en la enfermedad de la institución y de su preocupación por la contención de costes. Esto llevó a los participantes a movilizar estrategias por su cuenta para que el juego estuviera presente en sus prácticas y en su trabajo diario.

*Por parte de la institución, no tenemos ningún incentivo, tanto es así que somos nosotros los que vamos detrás de las cosas* (lúdicas)*, somos nosotros los que decoramos aquí, compramos todo con nuestro propio dinero. Sacamos dinero de nuestros bolsillos para que el ambiente sea un poco mejor.* (Muñeca)

*Yo entiendo que los técnicos de enfermería pueden utilizar el juego y las actividades lúdicas, pero aquí, en esta institución, no tenemos ese estímulo*. [...] *Creo que es más un problema de la cultura institucional, que se centra mucho en el proceso de la enfermedad, no tanto en el proceso del ser humano del niño, de la familia.* (Quemada)

*No hay estímulo por parte de la institución y no hay oferta de juegos o juguetes.* [...] *esto actuó demasiado como desincentivo* (para el uso del juego). (Casita)

Además de lo anterior, la mayoría de los entrevistados informó que la institución no había abordado el juego, las actividades lúdicas o la ludoteca en términos de formación continua u orientación para sus prácticas. También comentaron que su relación con las tecnologías y el entorno era intuitiva.

*Cuando empecé en pediatría, nunca recibí ninguna formación u orientación relacionada con el juego, la ludoteca o el uso de la ludoteca.* (Amiguita)

*Mira, llevo aquí un año y ocho meses, y hasta la fecha no he recibido ninguna formación. Aquí, los cursos están más enfocados a técnicas y procedimientos, pero no he visto esta parte sobre lúdica, juego, humanización.* (Saltando a la cuerda)

*Tenemos mucha formación sobre técnica, pero no sobre este tema que podría facilitar la técnica. Y creo que como mucha gente no lo pone en práctica, valdría la pena hacer este tipo de estímulo, porque no está estimulado, no está catalogado como algo importante.* (Elefantito de colores)

Así, el espacio físico de la unidad pediátrica, con la existencia de una sala de juegos/juguetería, fue elogiado por los participantes del estudio, contribuyendo para su adopción en sus prácticas, si así lo desearan.

*Creo que el espacio allí es muy bueno, es grande. A veces interactuamos con los niños jugando a la pelota allí, en el medio del pasillo, a veces en la sala de juegos*. (Saltitos)

*Se puede usar el juego y los juguetes en las habitaciones, en el patio, que tiene un área de juegos enorme, y en la sala de juegos también.* (Rayuela)

Ante este escenario, la ludoteca fue reconocida por los participantes como promotora de la incorporación de los recursos allí existentes en sus acciones profesionales, actuando en la recuperación del niño y haciendo menos traumática la experiencia de la internación, aunque su uso no fuera incentivado por la institución.

*Es un lugar* (la ludoteca) *que aporta ligereza a nuestro trabajo aquí. A veces entro allí, toco algo, cojo a un niño. Veo que transmite ligereza en un entorno pesado, como es el hospitalario. Es el único lugar donde recuerda que es una niña. Así que creo que la ludoteca desempeña un papel muy importante para dar un impulso al niño.* (Muñeca)

*No se anima a los técnicos a utilizar la ludoteca; la iniciativa la toma cada profesional individualmente. Algunos tienen más iniciativa y otros menos*. [...] *Creo que debería haber por lo menos una formación para el personal de enfermería que va a trabajar en pediatría o en cualquier lugar donde haya niños.* (Quemada)

## DISCUSIÓN

Los significados y las acciones del uso de las actividades lúdicas y del juego surgen y se configuran en el proceso interaccional desarrollado entre los técnicos de enfermería y los niños hospitalizados, y están implicados e influenciados por el contexto organizativo. Las perspectivas utilizadas para definir la práctica lúdica y el juego como elementos esenciales en la atención a los niños hospitalizados derivan de experiencias y creencias profesionales y personales, interiorizadas por los técnicos, que las relacionan con la humanización y la reducción de posibles traumas.

La hospitalización se considera una experiencia compleja para los niños, con potencial estresante y traumático, al igual que para sus cuidadores. Ambos están expuestos a un ambiente desconocido y ven alterada su rutina^(2,19)^. Este hecho fue relatado por la mayoría de los participantes en este estudio, que afirmaron que el juego es un mediador de experiencias más positivas durante la estancia del niño en el hospital. Esto demuestra la necesidad de que las instituciones inviertan en el ambiente de la unidad pediátrica para hacerla más acogedora y confortable para sus pacientes^(1)^ y, consecuentemente, promover y mantener una cultura de juego en la unidad.

Actualmente, se sabe que el juego y las actividades lúdicas confieren diversos beneficios durante la hospitalización del niño, entre ellos: minimizar los sentimientos de tristeza o traer alegría; ayudar a la recuperación del niño; facilitar la interacción con el profesional; minimizar el dolor y la incomodidad durante el procedimiento; provocar estímulos sensoriales; hacer que el niño esté más tranquilo y menos estresado; facilitar la aceptación del procedimiento; crear un vínculo; y fortalecer la confianza en el profesional^(11,20)^. Todas las novedades señaladas en la bibliografía también fueron comentadas por los participantes en este estudio.

Cabe destacar que el juego es un derecho del niño y le es inherente^(21)^. También es el lenguaje a través del cual expresan sus necesidades. Por lo tanto, promover cuidados que valoricen el juego ofrece oportunidades para la alegría de “ser niño”. Esto corrobora la opinión de los participantes en este estudio de que el niño, como persona, necesita una mirada diferente de la del adulto, con capacidad de revelar percepciones y necesidades. Para el equipo de enfermería, cuando los profesionales están dispuestos a jugar, además de atender a las necesidades del niño y fomentar la interacción, igualan el nivel de pensamiento del niño, construyendo y reconstruyendo significados y promoviendo cambios de comportamiento^(13)^, con el objetivo de proporcionar cuidados humanizados^(4)^. Entienden que este movimiento igualitario hace que la situación sea interesante para el niño, que convierte rápidamente esta oportunidad en una posibilidad de juego compartido con el profesional^(4,13)^. También permite a los niños expresar sus sentimientos y emociones, así como comprender mejor el proceso y la necesidad de hospitalización^(5,13)^_._

Según éste y otros estudios^(4,5,9)^, el uso del juego facilita también la realización de los procedimientos de enfermería, especialmente en técnicas como la punción y/o la administración de fármacos intravenosos, ya que los niños se tranquilizan y relajan y son capaces de cooperar más con el profesional. Además, estas prácticas promueven vínculos más estrechos, favorecen la recuperación y ayudan a aliviar la ansiedad y la tensión provocadas por la hospitalización^(5,9,22)^.

Por otro lado, algunos de los participantes entrevistados manifestaron que el uso de juegos y actividades lúdicas forma parte de las prácticas asistenciales de otros profesionales, y que su función es realizar procedimientos y cumplir prescripciones médicas. Esta actitud está en consonancia con los resultados encontrados en la literatura, que apuntan a la idea de que el juego es una actividad “extra” que no forma parte de la responsabilidad de la enfermería, y no cuenta entre sus competencias^(7,9,23)^. Por lo tanto, se observa que el juego no está incluido en la enfermería, teniendo un papel secundario e informal, voluntario y no institucionalizado, que no forma parte del proceso de atención de enfermería a los niños. Esto refuerza la necesidad de que los técnicos de enfermería resignifiquen el uso de estos recursos, provocando cambios actitudinales^(13)^ que favorezcan la asunción del juego como parte de la enfermería. Destaca el vínculo del profesional con el médico, a pesar de pertenecer al equipo de enfermería, siendo un aspecto interesante a explorar en futuros estudios. También cabe pensar que las prescripciones de enfermería no están dirigidas al juego.

En cualquier caso, hubo participantes que mostraron actitudes positivas hacia el juego. Como se muestra en otros estudios^(4,7,9,22)^, los técnicos de enfermería creen que estos recursos son beneficiosos durante la hospitalización del niño y, por lo tanto, han desarrollado la percepción de que son esenciales para la recuperación, el bienestar y la felicidad de sus pacientes^(24)^. Como tales, apoyan y defienden su utilización^(11)^, buscando sostenerlos, como aquellos que traen recursos para poder utilizarlos en su trabajo profesional, ya que no cuentan con todo el apoyo institucional para su incorporación. ¿Cómo las instituciones con unidades pediátricas están considerando el juego en sus agendas de costos?

Al discutir los factores que favorecen el uso del juego en las prácticas de los técnicos de enfermería, se observó que existen factores intrínsecos y extrínsecos para los profesionales^(9)^. Entre los factores intrínsecos están el favorecer la recuperación del niño y ayudarle a sobrellevar el estrés de la hospitalización, lo que le hace sentirse motivado y satisfecho. Según los entrevistados, estar alegre, divertirse y jugar no sólo aporta beneficios para el niño, sino también para ellos mismos, lo que fomenta aún más el uso de estas prácticas, un resultado similar al encontrado en la literatura^(7,9,22,23)^. También reconocen que cuando los niños juegan con las enfermeras, (re)significan su imagen como alguien más acogedor y juguetón^(7,13)^.

Los factores extrínsecos se asocian a la presencia de la ludoteca y de otros recursos en ella^(9)^. La ludoteca hospitalaria ayuda a los niños a superar el proceso de hospitalización dándoles confianza, facilitando su adaptación y minimizando su sufrimiento. Es un espacio donde pueden acercarse a su rutina fuera del hospital. Para el equipo de enfermería, puede ayudarles a acercarse al niño y a su familia, facilitando los procedimientos y pruebas, haciéndolos menos dolorosos y contribuyendo a la adherencia al tratamiento, lo que puede aumentar las posibilidades de recuperación del niño^(25)^. En este estudio, quedó claro que la existencia de un amplio espacio en la sala de pediatría fue un motivador para el uso de juegos y actividades lúdicas. Sin embargo, la falta de incentivos de la institución para explorar el entorno fue un factor desalentador para la participación en actividades lúdicas. Por lo tanto, está claro que las instituciones necesitan resignificar la pertenencia e inclusión del juego, la ludoteca y la ludoteca en el trabajo profesional del equipo de enfermería, con apoyo y reconocimiento efectivo de este tipo de intervención^(13)^.

Otro factor que actuó como desmotivador en este estudio fue la falta de inducción y orientación institucional, especialmente en lo que se refiere a proporcionar una formación profesional que abordara el juego y la ludoteca con niños, lo que les daría más seguridad y confianza para trabajar en pediatría. A esto se suman las deficiencias enfrentadas durante el curso técnico de enfermería, que les indicaba no establecer vínculos con sus pacientes. Este aspecto requiere una renovación y debería ser objeto de futuros estudios.

Otros estudios han mostrado que las exigencias del trabajo, la rutina de los procedimientos, el carácter mecánico de los cuidados, la falta de tiempo, la falta de infraestructura y de formación continuada han sido identificados como factores determinantes de la baja viabilidad de su utilización por el equipo de enfermería, cuestionándose el lugar del juego en la cultura institucional^(7,11,26)^. Además, otro estudio^(4)^ también reforzó estos hallazgos, revelando la no incorporación del juego en el proceso de enfermería en las instituciones, reforzando la falta de reconocimiento de la díada juego-cuidado, un avance que necesita convertirse en una agenda asistencial, especialmente en el ámbito pediátrico.

También es importante destacar la importancia del papel de la enfermera en las actitudes de su equipo. Deben ser entrenadas para desarrollar el juego y las actividades lúdicas de forma intencional, planificada e integrada en el plan de cuidados, así como incentivar y mantener a su equipo entrenado en el cuidado, llevándoles a cambiar los significados que hasta ahora atribuían a estas tecnologías, que, al ser experimentadas en las interacciones sociales, provocan cambios en el comportamiento^(7,13)^. Nuestro estudio demostró que la presencia de una enfermera que incentiva a su equipo hace que ellos se sientan más dispuestos y motivados a incorporar esos recursos en sus prácticas.

Por lo tanto, es evidente que las autoridades y los gestores hospitalarios deben ser conscientes de la importancia del juego y las actividades lúdicas para los niños hospitalizados, con el fin de dar su pleno apoyo a la implementación de actividades lúdicas y proporcionar los recursos necesarios para facilitar la integración y el uso del juego como una estrategia para promover la comodidad, aliviar la ansiedad y los temores, permitir la comunicación y la expresión de miedos, y promover la recuperación y el bienestar general del niño hospitalizado^(9)^. Estos cuidados institucionales crean una cultura y promueven, en las interacciones sociales^(13)^, la puesta en común de la singularidad de los recursos de humanización y satisfacción de necesidades, como el juego infantil.

Al dar voz a los técnicos de enfermería que trabajan en una unidad pediátrica, esta investigación pretende sensibilizar a los profesionales y gestores de estas instituciones sobre la importancia y los beneficios de la utilización del juego, con la posibilidad de provocar cambios y resignificaciones^(13)^ que promuevan la humanización de los cuidados pediátricos, con foco en esta categoría profesional. Las limitaciones de este estudio incluyen el hecho de que fue realizado en un único hospital, con una proporción significativa de los participantes que no tomaron parte en la segunda etapa de la investigación. Además, el hecho de que las entrevistas tuvieran lugar en persona y *en línea* también puede actuar como factor limitante, ya que existen diferentes mediaciones, exploraciones y presencias. Sin embargo, al ser una elección de los participantes, se cree que optaron por la forma con la que se sentían más cómodos, lo que favoreció las narraciones.

El marco de la IS fue poderoso para centrarse en los comportamientos de los participantes, señalando los significados que los estructuraban, así como los elementos de la escena social que movilizaban procesos en el *yo*. Las interacciones con el niño se destacaron en este contexto, así como el proceso de desarrollo de la conciencia de “sí mismo” como técnico de enfermería, a partir de un proceso de reflexividad que “cuestiona” lo “social” e institucionalmente impuesto a la profesión. Este proceso revela la emergencia del “yo” actuando en la autoconciencia, con aportes a la toma de decisiones sobre la incorporación del juego y lo lúdico.

## CONCLUSIÓN

Este estudio permitió conocer las percepciones de los técnicos de enfermería que trabajan en una unidad de hospitalización pediátrica sobre el uso del juego y las actividades lúdicas en sus prácticas profesionales. Hubo prevalencia del simbolismo de esos recursos contribuyendo a la ejecución de procedimientos técnicos y a la formación de vínculos. Hubo participantes que reconocieron y significaron los recursos como alineados al cuidado humanizado, con esfuerzos para invertir y mantener comportamientos que los incorporen.

La conclusión es que es necesario invertir en la discusión sobre la importancia de invertir en los técnicos de enfermería como categoría que puede contribuir y promover el juego durante la hospitalización. Para ello, es urgente renovar las posiciones institucionales, especialmente de los involucrados en la formación de estos profesionales, de sus empleadores y del consejo profesional. Los técnicos de enfermería pueden ampliar y sostener la presencia del juego y las actividades lúdicas en la vida cotidiana de los niños hospitalizados. El juego y el juguete son herramientas terapéuticas en este escenario; la dotación de infraestructura y apoyo para su uso generalizado es fundamental; y la formación profesional y continuada debe reconocer y abordar la cuestión dentro de lo que es pertinente a cada profesión. En cualquier caso, un buen comienzo es que los centros de formación técnica de enfermería aborden esta cuestión, desarrollando habilidades y competencias para su uso dentro de su ámbito profesional, con aportaciones a la calidad de los cuidados ofrecidos en las unidades hospitalarias pediátricas.

## Data Availability

El conjunto de datos que respalda los resultados de este estúdio no está disponible publicamente.
