# Supplementary material for: ERRATUM: Nursing technicians’ perceptions about the use of play and playfulness in professional practices
Source: Rev Esc Enferm USP. 2025 Aug 11;59:e20250024er. doi: 10.1590/1980-220X-REEUSP-2025-0024eren (PMC12356576; doi:10.1590/1980-220X-REEUSP-2025-0024eren)
Supplement: Translation

# Translation: ERRATA: Percepções de técnicas de enfermagem acerca do uso do lúdico e do brincar nas práticas profissionais

No artigo “Percepções de técnicas de enfermagem acerca do uso do lúdico e do brincar nas práticas profissionais”, com número de DOI: https://doi.org/10.1590/1980-220X-REEUSP-2025-0024pt, publicado na Revista da Escola de Enfermagem da USP, 59:e20250024, 2025, na página 1:

**Onde se lia:**

Gabriele Petruccelli^1^

Monika Wernet^2^

Aline Oliveira Silveira^3^

Edmara Bazoni Soares Maia^2^

Cristiane Leite de Almeida^4^

Fernanda Manuela Loureiro^5^

^1^ Universidade Federal de São Carlos, Programa de Pós-Graduação em Enfermagem, São Carlos, SP, Brasil.

^2^ Universidade Federal de São Carlos, Departamento de Enfermagem, São Carlos, SP, Brasil.

^3^ Universidade de Brasília, Departamento de Enfermagem, Brasília, DF, Brasil.

^4^ Escola Técnica Estadual Paulino Botelho, São Carlos, SP, Brasil.

^5^ Universidade Católica Portuguesa, Lisboa, Portugal.

**Leia-se:**

Gabriele Petruccelli^1^

Monika Wernet^2^

Aline Oliveira Silveira^3^

Edmara Bazoni Soares Maia^4^

Cristiane Leite de Almeida^5^

Fernanda Manuela Loureiro^6^

^1^ Universidade Federal de São Carlos, Programa de Pós-Graduação em Enfermagem, São Carlos, SP, Brasil.

^2^ Universidade Federal de São Carlos, Departamento de Enfermagem, São Carlos, SP, Brasil.

^3^ Universidade de Brasília, Departamento de Enfermagem, Brasília, DF, Brasil.

^4^ Universidade Federal de São Paulo, Escola Paulista de Enfermagem, São Paulo, SP, Brasil.

^5^ Escola Técnica Estadual Paulino Botelho, São Carlos, SP, Brasil.

^6^ Universidade Católica Portuguesa, Lisboa, Portugal.

# Translation: ERRATA: Percepciones de las técnicas de enfermería sobre el uso del juego y el entretenimiento en las prácticas profesionales

En el artículo “Percepciones de las técnicas de enfermería sobre el uso del juego y el entretenimiento en las prácticas profesionales”, con número de DOI: https://doi.org/10.1590/1980-220X-REEUSP-2025-0024es, publicado en la Revista da Escola de Enfermagem da USP, 59: e20250024, 2025, en la página 1:

**Donde se leía:**

Gabriele Petruccelli^1^

Monika Wernet^2^

Aline Oliveira Silveira^3^

Edmara Bazoni Soares Maia^2^

Cristiane Leite de Almeida^4^

Fernanda Manuela Loureiro^5^

^1^ Universidade Federal de São Carlos, Programa de Pós-Graduação em Enfermagem, São Carlos, SP, Brasil.

^2^ Universidade Federal de São Carlos, Departamento de Enfermagem, São Carlos, SP, Brasil.

^3^ Universidade de Brasília, Departamento de Enfermagem, Brasília, DF, Brasil.

^4^ Escola Técnica Estadual Paulino Botelho, São Carlos, SP, Brasil.

^5^ Universidade Católica Portuguesa, Lisboa, Portugal.

**Lea:**

Gabriele Petruccelli^1^

Monika Wernet^2^

Aline Oliveira Silveira^3^

Edmara Bazoni Soares Maia^4^

Cristiane Leite de Almeida^5^

Fernanda Manuela Loureiro^6^

^1^ Universidade Federal de São Carlos, Programa de Pós-Graduação em Enfermagem, São Carlos, SP, Brasil.

^2^ Universidade Federal de São Carlos, Departamento de Enfermagem, São Carlos, SP, Brasil.

^3^ Universidade de Brasília, Departamento de Enfermagem, Brasília, DF, Brasil.

^4^ Universidade Federal de São Paulo, Escola Paulista de Enfermagem, São Paulo, SP, Brasil.

^5^ Escola Técnica Estadual Paulino Botelho, São Carlos, SP, Brasil.

^6^ Universidade Católica Portuguesa, Lisboa, Portugal.
